# Uptake of H^14^CO_3_^−^/^14^CO_3_^2−^ by calcite: impact of ISA and chloride

**DOI:** 10.1039/d5ra05547d

**Published:** 2025-10-15

**Authors:** Rosa Ester Guidone, Nils Huber, Frank Heberling, Thomas Sittel, Natalia Palina, Florian Bocchese, Stéphane Brassinnes, Marcus Altmaier, Xavier Gaona

**Affiliations:** a Karlsruhe Institute of Technology (KIT), Institute for Nuclear Waste Disposal (INE) Hermann-von-Helmholtz-Platz 1 76344 Eggenstein-Leopoldshafen Germany rosa.guidone@kit.edu Xavier.Gaona@kit.edu; b ONDRAF/NIRAS, Belgian Agency for Radioactive Waste and Enriched Fissile Materials Brussels Belgium

## Abstract

The uptake of inorganic ^14^C (H^14^CO_3_^−^/^14^CO_3_^2−^) by calcite was investigated at pH ≈ 8.3 in the absence and presence of isosaccharinate ([ISA]_tot_ = 10^−5^–0.2 M) and chloride ([NaCl]_tot_ = 10^−4^–2.0 M). Calcite is representative of the last degradation stage of Portland cement, whereas ISA and chloride are components expected in low- and intermediate level short-lived waste (L/ILW-SL). A moderate ^14^C uptake is observed at short contact times, followed by a steady increase of the retention with time resulting in distribution ratios ≈ 10^3^ L kg^−1^ at *t* = 60 days. This is explained by fast adsorption, followed by incorporation into the calcite structure through recrystallization phenomena. The minor impact of ISA and chloride on ^14^C uptake is possibly related to the formation of Ca-ISA complexes and the alteration of the surface properties of calcite. The modelling of the recrystallization process allows long-term predictions of the ^14^C uptake in the context of repositories for L/ILW-SL.

## Abstract 2

Retention of inorganic ^14^C by calcite quantified and mechanistically assessed under advanced cement degradation conditions.

## Graphical abstract

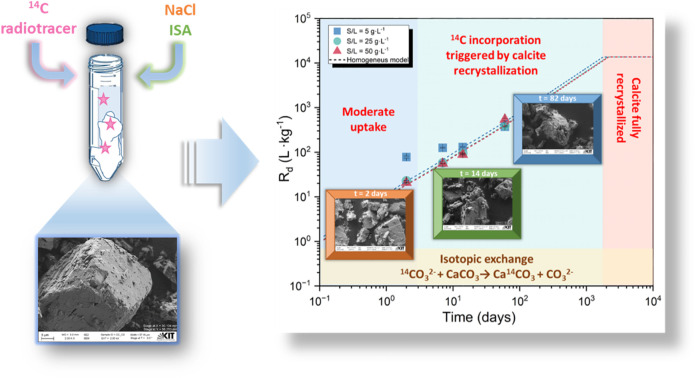

## 1 Introduction

The radioactive isotope ^14^C (*t*_1/2_ = 5700 a) is found in very low concentrations in nature (≈1 ppt), where it is primarily produced in the lower atmosphere layers by ^14^N neutron activation triggered by cosmic rays. In nuclear reactors, ^14^C is produced by the activation of stable parent isotopes (^14^N, ^13^C and ^17^O) present in *e.g.*, core structural materials, fuel and reactor coolant.^1,2^ In the resulting radioactive waste, ^14^C can be present as organic compounds (*e.g.*, small carboxylic acids, alcohols, aldehydes or methane) and inorganic carbonate, although the latter is expected to prevail in (near-surface) repositories for low- and intermediate level short-lived waste (L/ILW-SL).^2^

Cementitious materials are extensively used in L/ILW repositories for construction purposes and as a relevant component in the engineered barrier system. Degradation processes determined by the contact with groundwater (as well as surface water in near-surface disposal concepts) will affect the composition of hydrated cement. Degradation is generally discussed as a sequence of four degradation stages (I to IV), although this sequence is also influenced by the cement type and the use of SI such as fly ash or blast furnace slag. Degradation stage I is dominated by the dissolution of Na and K hydroxides and oxides, resulting in pH > 13. Under these conditions, portlandite solubility is low and calcium silicate hydrate (C–S–H) phases are defined by high Ca/Si ratio. In the degradation stage II, the pore water composition is controlled by portlandite solubility, which defines pH ≈ 12.5 and [Ca] ≈ 20 mM. After the complete dissolution of portlandite, the degradation stage III is dominated by the incongruent dissolution of C–S–H, AFm and AFt phases and the decrease of pH from ≈ 12.5 to ≈ 10.^3,4^ In the last degradation state (IV), C–S–H phases are completely dissolved and the remaining aggregate minerals (*e.g.*, calcite) control the composition and pH (<10) of the pore solution. Calcite is thus expected as one of the main solid phases responsible for the retention of radionuclides in advanced stages of cement degradation. Although other secondary phases (*e.g.*, SiO_2_, traces of C–S–H phases with low Ca/Si ratio, *etc.*) cannot be excluded at this stage,^5^ calcite is expected to control the inorganic carbonate concentration in solution (mainly constitute by HCO_3_^−^ aqueous species) and pH with a value of ≈ 8.3.^2,6^ Note also that in near-surface disposal concepts, accelerated cement carbonation (and thus calcite formation) needs to be accounted for in specific scenarios. This aspect is of relevance when assessing the retention of ^14^CO_3_^2−^, with *t*_1/2_ = 5700 a.

Calcium carbonate (CaCO_3_) exists in several polymorphs (calcite, aragonite and vaterite) or even hydrated forms (hydrated ikaite, monohydrocalcite) depending on the conditions.^7^ Calcite is the thermodynamically most stable polymorph of CaCO_3_ at ambient conditions, and it occurs in several geological formations (chalk, limestone, travertine, calcareous sandstone *etc.*).^8^ Recrystallization processes can lead to the incorporation of other ions into the calcite structure, as well as to the isotopic exchange with other Ca and C isotopes present in solution. Heberling and co-authors investigated the interlink between recrystallization rates, morphology and particle size of seven calcite samples, from coarse (surface area 0.2 m^2^ g^−1^) to fine grained (surface area 23.8 m^2^ g^−1^) material following the isotopic exchange with ^45^Ca.^9^ The authors concluded that calcite recrystallization is an interfacial free energy driven Ostwald-ripening process, in which particle roughness effects dominate over the effect of crystal habitus and particle size. Allard and co-authors^10^ investigated the uptake of ^14^CO_3_^2−^ by natural calcite (S/L = 20 g L^−1^) in artificial granitic pore water (pH = 8.0–8.2) by means of batch sorption experiments. The authors observed a steady increase of ^14^C uptake as function of time with distribution coefficient *R*_d_ ≈ 80 L kg^−1^ at *t* = 185 days, and proposed isotopic exchange as main retention mechanism. Boylan and co-workers investigated the retention of ^14^CO_3_^2−^ following (i) the precipitation of calcite in supersaturation solutions, and (ii) by isotopic exchange with crystalline calcite at S/L = 5, 20 and 50 g L^−1^.^11^ In the experiments conducted in the presence of calcite, the authors observed a minor increase of the uptake of ^14^CO_3_^2−^ within the monitored time window (*t* = 7–28 days) with *R*_d_ ≈ 5 L kg^−1^.

Large quantities of cellulose-based materials (*e.g.*, paper, wood, textile, *etc.*) are present in L/ILW-SL. Under the alkaline conditions imposed by cementitious environments, cellulose undergoes degradation resulting in the formation of α-isosaccharinic acid (ISA) as main degradation product.^12–14^ ISA has been shown to form strong complexes with hard Lewis acids such as actinides, lanthanides and some transition metals,^15–19^ but forms also weaker complexes with Ca under near-neutral to hyperalkaline pH conditions.^20–22^ Although ISA is not expected to interact with H^14^CO_3_^−^/^14^CO_3_^2−^, the impact of this organic ligand on the solubility and surface properties of calcite may affect the retention of this radionuclide. High chloride concentrations (up to ≈ 4–5 M) are reported for specific waste streams originating from evaporator concentrates in nuclear power plants.^23^ These high chloride concentrations can affect the retention of radionuclides in cementitious environments, particularly for anionic radionuclides like CO_3_^2−^, for which competition effects may become relevant.

In this context, this work aims at the quantitative description of H^14^CO_3_^−^/^14^CO_3_^2−^ uptake by calcite under ambient atmospheric conditions. Moreover, the impact of ISA and chloride on the retention of H^14^CO_3_^−^/^14^CO_3_^2−^ is investigated by means of systematic batch sorption experiments on the ternary and quaternary systems calcite-^14^C-ISA, calcite-Cl-^14^C, and calcite-Cl-^14^C-ISA, respectively. Calcite recrystallization and its potential impact on the retention of ^14^C is addressed with a dedicated series of inactive batch experiments coupled with modeling approaches previously described in the literature.^9,24^

## 2 Experimental

### 2.1 Chemicals and analytical methods

Milli-Q water (Millipore, 18.2 MΩ cm) was used for the preparation of aqueous solutions. All samples were prepared and equilibrated at *T* = (22 ± 2) °C under air atmosphere. Active experiments were conducted in dedicated air-gloveboxes. CaCO_3_ was purchased from Sigma-Aldrich (ACS reagent, 99.0%). NaCl EMSURE (≥99.5%), isosaccharinic acid −1, 4-lactone (ISA_L_) (98.0%), and NaOH (and HCl) Titrisol were obtained from Supelco, Biosynth and Merck, respectively. Sorption experiments were performed using ^14^CO_3_^2−^ as radiotracer. Two ^14^CO_3_^2−^ (*t*_1/2_ = 5700 a) stock solutions were purchased from Eckert & Ziegler Nuclitec GmbH with a purity of > 99.9%: (i) a ^14^CO_3_^2−^ solution in Na_2_CO_3_ as inactive carrier, which was diluted in Milli-Q to obtain the target concentrations of [^14^CO_3_^2−^] = 1.6 × 10^−7^ M (3.75 kBq mL^−1^) and [NaCO_3_] = 4.7 × 10^−3^ M; (ii) a ^14^CO_3_^2−^ solution in 0.1 M NaOH and containing as inactive carrier [Na_2_CO_3_] = 2.8 × 10^−4^ M, was diluted (1 : 100) in calcite saturated pore solution to achieve [^14^CO_3_^2−^] = 2.4 × 10^−7^ M (5.58 kBq mL^−1^). Note that at the pH of the experiments (≈8.3), carbonate is predominantly found as HCO_3_^−^ (*ca.* 96%). Keeping this in mind and for the sake of simplicity, the notation ^14^C has been used throughout the text instead of referring to H^14^CO_3_^−^/^14^CO_3_^2−^.

The measurement of pH was performed using combination pH-electrodes (Orion Ross, Thermo Scientific) filled with 3.0 M KCl freshly calibrated against standard pH buffers (pH = 7–10, Merck).

The measured pH value in aqueous solutions at *I*_m_ ≥ 0.1 mol kg^−1^, pH_exp_, is an operational apparent value related to the molal proton concentration ([H^+^], in mol kg^−1^) by pH_m_ = pH_exp_ + *A*_m_, where pH_m_ = −log [H^+^] and *A*_m_ is an empirical parameter including the activity coefficient of the proton, γ(H^+^), and the liquid junction potential of the electrode for a given background electrolyte, ionic strength, temperature and pressure. The values of *A*_m_ for NaCl solutions were taken from the literature.^25^

### 2.2 Calcite saturated solution

Calcite saturated solution (CSS) was prepared by equilibrating CaCO_3_ with Milli-Q water (S/L = 1 g L^−1^) under air. The suspension was mixed with a magnetic stirrer (100 rpm) at room temperature for at least 24 hours. Total inorganic carbon content (TIC) of CSS was determined with a Shimadzu (TOC-L) device. To remove possible solid residues in the supernatant solution, the CSS was centrifuged at 6000 g for 10 min (Hettich Zentrifugen, EBA 280), or otherwise separated by ultrafiltration using 10 kDa filters (2–3 nm cut-off Nanosep®, Pall Life Sciences). The total calcium concentration in inactive and active samples was determined by inductively coupled plasma-optical emission spectroscopy (ICP-OES, PerkinElmer, Optima8300) and ICP-MS (Inductively Coupled Plasma-Mass Spectrometry, Nexion, 2000P), respectively.

Chloride concentration in CSS was determined by ion chromatography (IC) using a Thermo Fisher (Dionex) ICS-3000 device. This concentration was used as lower limit in the ^14^C sorption experiments under varying NaCl concentration as described in Section 2.5. The composition of CSS is reported in Table 1, as determined experimentally in this work and calculated using thermodynamic data selected in the ThermoChimie database v.12a.^26^

**Table 1:** Composition of the calcite saturated solution (CSS) under air atmosphere used in this work. Uncertainties are reported as two times the standard deviation (2*σ*) of mean values. Values in cursive correspond to the CSS composition as calculated using thermodynamic data selected in the ThermoChimie database v.12a.^26^*C*_tot_ indicates the total carbonate concentration expressed as [HCO_3_^−^] + [CO_3_^2−^]

pH	[Ca]_tot_ (M)	*C*_tot_ (M)	[Cl]_tot_ (M)
(8.3 ± 0.1)	(5.79 ± 0.27) × 10^−4^	(1.21 ± 0.10) × 10^−3^	(8.40 ± 0.10) × 10^−5^
8.3	5.0 × 10^−4^	1.0 × 10^−3^	

### 2.3 Characterization of calcite recrystallization: XRD, BET and SEM measurements

Calcite recrystallization was studied with two independent systems, *i.e.*, in the absence and presence of 2.0 M NaCl. Batch experiments were prepared by contacting 5 g of calcite with CSS at a solid-to-liquid ratio (S/L) of 25 g L^−1^. Independent batch samples were equilibrated under continuous stirring (100 rpm) for three contact times, *i.e.*, *t* = 2, 14 and 82 days. After the corresponding contact time, solid phase separation was achieved by vacuum filtration through 0.45 μm nylon filters. The resulting wet material was dried in a desiccator for 14 days, and characterized by X-ray powder diffraction (XRD), Brunauer, Emmett and Teller method (BET), and focus ion beam – scanning electron microscopy (FIB-SEM).

XRD measurements were carried out to gain insight on the crystal structure and crystallite size of calcite. Measurements were been performed with a Bruker D8 Advance X-ray powder diffractometer (CuKα radiation, *λ* = 1.54184 Å) in the interval 2*θ* = 2–65°, with incremental steps of 0.012° (2*θ*°) and a total counting time of 2.0 s per step.

The specific surface area (*A*_s_) of the calcite material after different contact times was determined from gas (N_2_) adsorption experiments following the BET method.^27^ N_2_-BET measurements were performed on Quantachrome NOVA 4000e. Samples were dried at 105 °C under vacuum for *ca.* 17 hours before BET measurements.

FIB-SEM images were recorded using a Zeiss FIB-SEM device (CrossBeam 350 KMAT). A secondary electron SE detector (Everhart-Thornley) was used for the characterization of the calcite morphology. FIB-SEM samples were prepared on an Al stab and fixed with a double sided adhesive C-tab.

### 2.4 Stability of ISA in calcite saturated solution

A stock solution with 0.1 M ISA was prepared by dissolving 0.16 g of isosaccharinic acid −1,4-lactone (ISA_L_) in 10 mL of 0.2 M NaOH. The solution was equilibrated for 24 hours to trigger the opening of the ISA carbon chain. 0.5 mL of 0.1 M ISA were added to 4.5 mL of CSS to obtain [ISA]_tot_ = 10 mM. The excess of NaOH was titrated to pH ≈ 8.3 with 0.1 M HCl. The resulting solution was stored under air atmosphere, and characterized at different contacting times (*t* = 2, 7, 14, 30, 60, 160 days) by ^1^H and ^13^C NMR (Nuclear Magnetic Resonance) analysis. NMR analysis was carried out with a Bruker Avance III 400 spectrometer, operating at 400.18 MHz for ^1^H and 100.63 MHz for ^13^C. NMR spectrometer is equipped with a 5 mm inverse broadband probe-head furnished with z-oriented magnetic field gradient capability. In each measurement, the magnetic field was stabilized by locking it to the ^2^D signal of the solvent. For the individual samples, 32–64 scans were taken to record ^1^H NMR spectra with the WATERGATE pulse program. The pH was also monitored as a function of time.

### 2.5 Sorption experiments in the absence and presence of ISA and chloride

Sorption experiments were conducted in 15 mL polypropylene vials (Sarstedt) and prepared by contacting fresh calcite with CSS. The suspensions were equilibrated for 1 day before further use. The effect of solid-to-liquid ratio on the uptake was evaluated with independent batch sorption experiments containing [^14^C]_tot_ = 1.7 × 10^−9^ M (38.5 Bq mL^−1^) and varying S/L = 5, 25 and 50 g L^−1^. For these samples, the evolution of the activity of ^14^C in the aqueous phase was monitored at 2 ≤ *t* (days) ≤ 60. The uptake of ^14^C by calcite was also investigated as a function of the initial ^14^C concentration (sorption isotherms), with 8.6 × 10^−11^ M ≤ [^14^C]_tot_ ≤ 8.6 × 10^−9^ M, S/L = 5 and 25 g L^−1^ and 2 ≤ *t* (days) ≤ 180.

The effect of ISA on the retention of ^14^C by calcite was investigated as a function of S/L and ISA concentration. In all cases, ISA was added after a pre-equilibration of the suspension containing calcite, CSS and ^14^C for two days. A first series of experiments was conducted at [^14^C]_tot_ = 1.7 × 10^−9^ M (38.5 Bq mL^−1^), [ISA]_tot_ = 10^−2^ M, S/L = 5, 25, 50 g L^−1^, and the activity of ^14^C was monitored at 5 ≤ *t* (days) ≤ 52. A second series of experiments explored the retention of ^14^C with increasing ligand concentration, with [^14^C]_tot_ = 8.6 × 10^−9^ M (20 Bq mL^−1^), S/L = 5, 25 g L^−1^ and 10^−4^ M < [ISA]_tot_ < 0.2 M. The ^14^C concentration was monitored at 12 ≤ *t* (days) ≤ 289. A pH correction was required in samples with high ISA concentration due the alkaline pH (≈12) of the ISA stock solutions.

The effect of chloride on the retention of ^14^C was investigated at NaCl concentrations of 1 × 10^−4^, 3 × 10^−3^, 3 × 10^−2^, 0.3 and 2.0 M. The suspensions containing calcite, CSS and NaCl were equilibrated for 3 days before the addition of the ^14^C tracer. A systematic increase of pH was observed in 0.3 and 2.0 M NaCl solutions, which required a correction to the target pH value of ≈ 8.3. Sorption experiments were conducted with [^14^C]_tot_ = 8.6 × 10^−9^ M (20 Bq mL^−1^) and S/L = 5, 25 g L^−1^.

The combined effect of chloride and ISA on the retention of ^14^C was studied with a series of independent batch experiments containing [^14^C]_tot_ = 8.6 × 10^−9^ M (20 Bq mL^−1^), [ISA]_tot_ = 1 × 10^−2^ M, 10^−4^ M < [NaCl]_tot_ < 2.0 M, and S/L = 5, 25 g L^−1^. The suspensions of calcite, CSS and NaCl were equilibrated for 3 days before the addition of ^14^C. In a last step, ISA was added after a contact time of 2 days.

The ^14^C activity was quantified after phase separation by means of liquid scintillation counting. Phase separation was achieved by ultracentrifugation (90 000 rpm) using an OPTIMA XPN-QC ultracentrifuge and rotor type 90Ti (both Beckman–Coulter). Volumes of *ca.* 4.2 mL were transferred into polypropylene tubes (Beckman–Coulter) and heat-sealed afterwards using a tube topper device (Beckman–Coulter, Tube Topper Kit, 60 Hz). LSC measurements were performed using a Tri-Carb 3110 TR. LSC samples were prepared as follows: a variable sample volume (0.1–1 mL) was mixed with 10 mL of scintillator solution (Hionic-Fluor, PerkinElmer) and 1.5 mL of 2 M NaOH. The latter is required to avoid the loss of ^14^C through degassing as ^14^CO_2_ due to the acidity of the LSC cocktail. To determine the absolute sample activity (DPM), the measured activity (CPM) was corrected using the appropriate counting efficiency, as described in the SI. The detection limit for ^14^C determined by LSC was ≈ 0.6 Bq mL^−1^ (2.6 × 10^−11^ M).

The retention of ^14^C by calcite was evaluated in terms of distribution ratios (*R*_d_, in L kg^−1^) as a function of equilibration time, S/L, [ISA]_tot_ and [NaCl]_tot_. *R*_d_ values are calculated as described in eqn (1):1
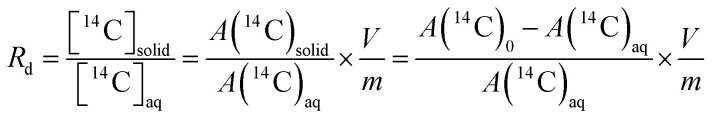
where *A*(^14^C)_0_ is the initial ^14^C activity, *V* is the volume of the solution sample (L) and *m* is the mass of calcite (kg). Most *R*_d_ values were quantified by measuring *A*(^14^C)_0_ and *A*(^14^C)_aq_ (activity in the aqueous phase, after phase separation). Furthermore, for selected systems, *R*_d_ values were also quantified by measuring ^14^C activity in the suspension (aqueous and solid phase) and in the aqueous phase, which reflect the first term of the eqn (1). Both approaches resulted in consistent distribution ratios. Uncertainties in *R*_d_ values were calculated as two times the standard deviation of three sample replicates.

### 2.6 Determination of recrystallization rates

Calcite recrystallization was monitored through the decrease of ^14^C activity in solution as a function of the contact time. The fraction of calcite recrystallized was evaluated using the homogeneous and heterogeneous recrystallization models. Both models were initially adapted by^24,28^ from the incorporation models described by^29^ and.^30^ The common assumption in both models is that the ratio of radiotracer (^14^C) to the carrier element (inactive C) is at any time uniform in the newly formed solid, and equal, to that in solution, see eqn (2). Additionally, as the reaction occurs at the saturation equilibrium of calcite, *m*(C)_tot_ (total aqueous carbonate concentration, in mol kg^−1^) is assumed to be constant throughout the experiments.2
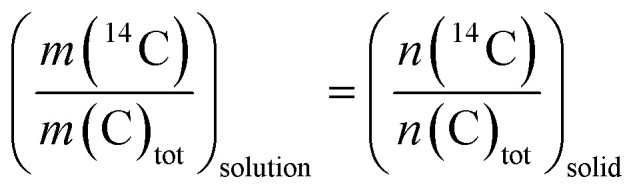
where *m*(^14^C) (mol kg^−1^) and *n*(^14^C) (mol) represent the molality and moles of the radiotracer in solution and the solid phase, respectively.

In the heterogeneous model, the fraction of solid phase recrystallized does not contribute to any further solid-solution equilibration. As a consequence of the heterogeneous model the ratio *n*(^14^C)*/n*(C)_tot_ decreases throughout the experiment. Instead, the homogenous model predicts that once a portion of calcite is recrystallized, it is assumed to recrystallize repeatedly over time, maintaining the partial equilibrium between the solution and the calcite that has taken part in the recrystallization process. The moles of recrystallized calcite for the heterogeneous and homogeneous models are calculated according to eqn (3) and (4), respectively:3
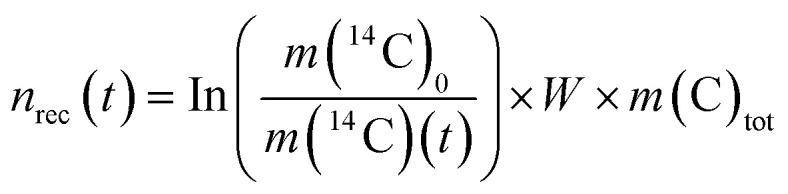
4
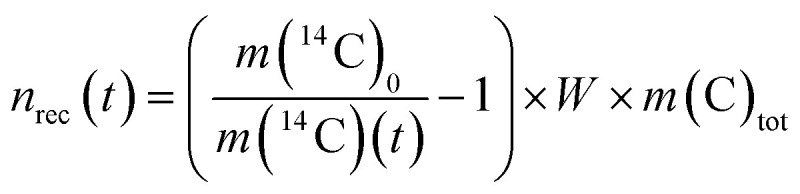
where *n*_rec_(*t*) (mol) are the moles of recrystallized calcite at given time *t* (s), *W* (kg) is the weight of the solution, *m*(^14^C)_0_ (mol kg^−1^) is the initial molality of ^14^C, *m*(^14^C)(*t*) is the molality of the radiotracer at time *t* and *m*(C)_tot_ is the total molality of C in the aqueous phase. It is further assumed that calcite with a natural carbon isotopic composition (>99% ^12^C) and Ca^14^CO_3_ form an ideal solid solution and that carbon isotope fractionation effects can be neglected during close-to-equilibrium recrystallization.

The recrystallization rates normalized by the surface area *A*_s_ (m^2^ kg^−1^) for the experimental time interval Δ*t* = *t*_i_ − *t*_*i*−1_ (s), are determined as described in eqn (5):5
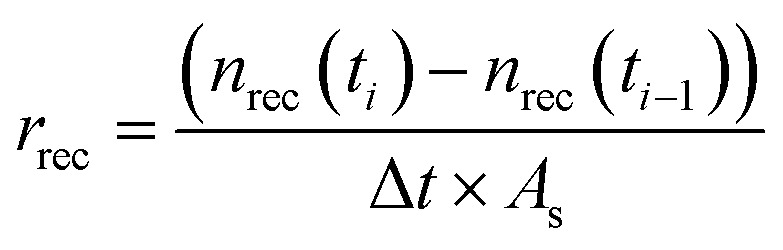
where *r*_rec_ is the recrystallization rate (mol (s^−1^ m^2^)) and *n*_rec_(*t*_*i*_) *– n*_rec_(*t*_*i*−1_) (mol) is the difference of the moles of recrystallized calcite in the interval of time *Δt* (s). The surface area was quantified for analogous inactive systems at the same contact times as in the active experiments (see Section 2.3).

## 3 Results and discussion

### 3.1 Calcite recrystallization

**Fig. 1:**
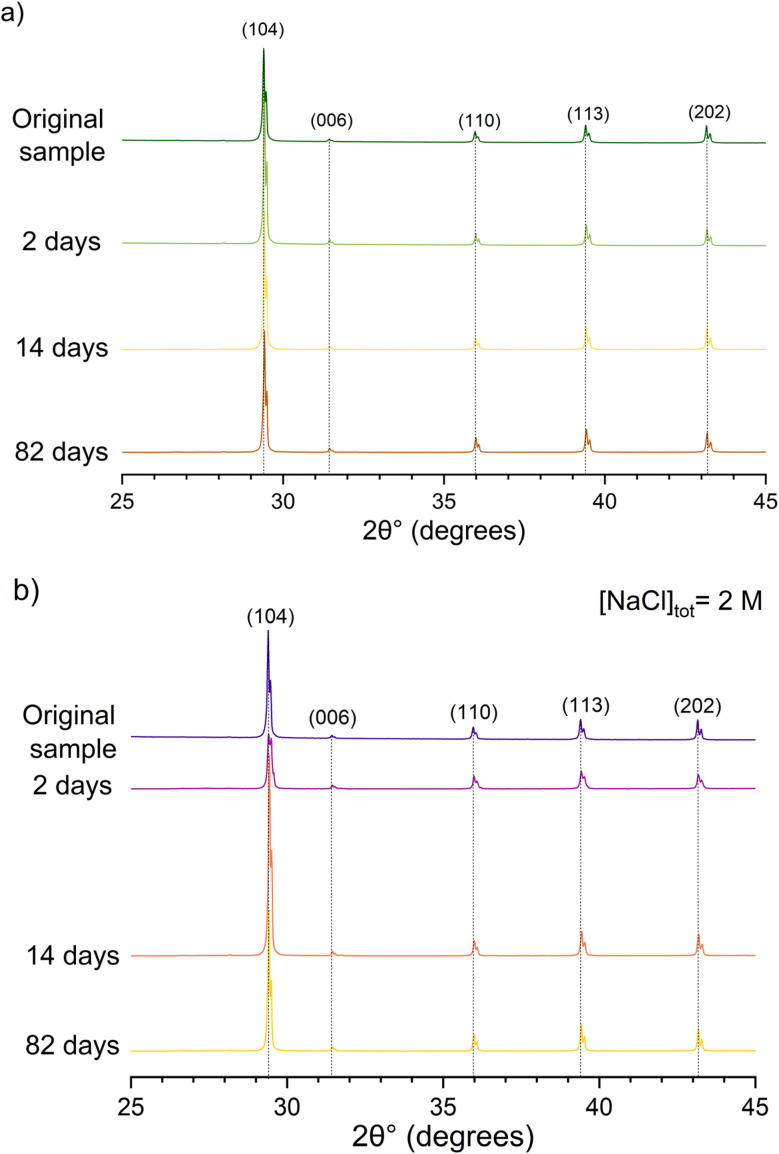
XRD spectra of calcite samples equilibrated in calcite saturated pore water in the (a) absence and (b) presence of [NaCl]_tot_ = 2 M at different contacting times, *t* = 2, 14 and 82 days. The XRD diffractogram of the original calcite material is reported for comparison purposes.

The diffractogram of the original calcite material shown in Fig. 1a is defined by the characteristic diffraction patterns of calcite, with a main feature at 2*θ*° = 29.42° (*d*_(104)_ = 3.03 Å). The experimental diffraction peaks fit well with the reference data reported for synthetic calcite (PDF 05-0586). The two planar distances at 2*θ*° = 64.67° (*d*_(300)_ = 1.44 Å) and at 2*θ*° = 31.46° (*d*_(006)_ = 2.84 Å) confirm rhombohedral crystal configuration (*R*3̄*c*) with the unit cell parameter: *a* = 4.99 Å and *c* = 17.05 Å. No further phases are detected. A more comprehensive analysis of the XRD data of calcite, prior to equilibration, is reported in the SI (Fig. S1). The crystalline structure (Fig. 1a) and of the three calcite samples equilibrated at the contacting times, *t* = 2, 14 and 82 days, is not affected by the recrystallization process.

Fig. 1b shows the diffractograms of calcite samples equilibrated in the presence of 2.0 M NaCl. Calcite is identified as main solid phase, together with traces of NaCl (PDF 05-0628) from adherent NaCl matrix solution. The crystallite size as determined with the Debye–Scherrer method is comparable to the one determined for the NaCl-free system.

The SEM-micrographs and BET specific surface areas of the original calcite material, and samples equilibrated for *t* = 2, 14 and 82 days are reported in Fig. S2–S4, Fig. S5–S7 (SI) and Table 2, respectively. SEM images of the original calcite material indicate rhombohedral shaped calcite particles, with planar faces and the presence of some surface roughness. SEM pictures reflect also the heterogeneous distribution in the calcite particle size, with large crystals (40–70 μm) but also abundant smaller particles (≤500 nm). The comparison of the samples at 2 days and 82 days of contacting time, shown in Fig. S2 (SI), qualitatively suggest a decrease of the fraction of smaller particles with increasing contacting time. Sharp interfacial edges were observed for the original calcite material and for the sample equilibrated for 2 days of contacting time. For longer equilibration times (*t* > 2 days), calcite surfaces become irregular and signs of dissolution processes become more evident (see regions marked in Fig. S3 and S4 in SI).

**Table 2:** Time evolution of specific surface area (*A*_s_) of calcite in the absence and presence of chloride ([NaCl] = 2 M), as quantified by BET measurements

Time, (days)	*A*_s_, (m^2^ g^−1^)	*A*_s_, (m^2^ g^−1^) [NaCl] = 2 M
—	(0.56 ± 0.05)	
2	(0.59 ± 0.05)	(0.40 ± 0.05)
14	(0.47 ± 0.05)	(0.30 ± 0.05)
82	(0.40 ± 0.05)	(0.23 ± 0.05)

Both the original calcite material and the sample equilibrated for two days are characterized by a similar surface area (*A*_s_ ≈ 0.6 m^2^ g^−1^), which is also comparable with the *A*_s_ of calcite sample labelled as “Aged Coarse Cc” in Heberling *et al.*^9^ The surface area decreases successively as a function of equilibration time, with *A*_s_ = 0.47 and 0.40 m^2^ g^−1^ at *t* = 14 and 82 days of equilibration time, respectively. The observed decreasing trend of *A*_s_ is clearly beyond the uncertainty of the method and can be explained by the increase of the particle size (Fig. S2, SI). This effect is attributed to the Ostwald ripening, particularly affecting to the smaller particles in the calcite material. The SEM micrographs collected for the calcite system in 2 M NaCl show also particles with a heterogeneous size distribution, characterized by sharp interfacial edges and partially smooth interfaces (Fig. S5–S7 in SI). BET surface area of calcite in 2 M NaCl decreases with time (Table 2), although with slightly lower absolute values compared to chloride-free systems.

### 3.2 ^14^C retention by calcite

#### 3.2.1 Uptake of ^14^C in the absence of ISA and NaCl

**Fig. 2:**
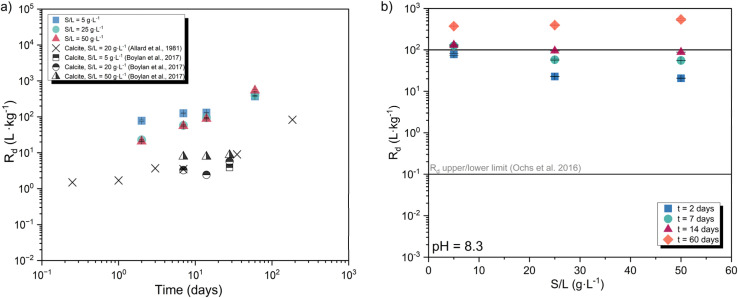
(a) Sorption kinetics for the uptake of ^14^C uptake by calcite for three systems with S/L = 5, 25 and 50 g L^−1^, obtained in the time frame 2 ≤ *t* (days) ≤ 60. Cross shaped symbols represent the kinetic sorption experiments (S/L = 20 g L^−1^) carried out by Allard *et al.*^10^ and half-filled squares, circles and triangles symbols show the sorption results reported by Boylan *et al.*^11^ (b) Distribution ratios *R*_d_ determined in this work for the uptake of ^14^C by calcite as a function of S/L. Solid lines represent the upper and lower *R*_d_ limits reported by Ochs *et al.*^2^

Fig. 2a and b show the kinetics of the ^14^C uptake by calcite at S/L = 5, 25 and 50 g L^−1^. The uptake increases steadily with time for all three systems, reaching *R*_d_ ≈ 5 × 10^2^ L kg^−1^ at *t* = 60 days (Fig. 2a). The slow but steady increase of ^14^C uptake with time is attributed to isotopic exchange with ^12^C in calcite, triggered by calcite recrystallization. A similar time-dependency of the ^14^C uptake by calcite was previously reported by Allard *et al.* (see Fig. 2a), although with overall lower *R*_d_ values.^10^ Allard and co-workers performed batch sorption experiments using artificial granitic groundwater with pH = 8.0–8.2, [Ca]_tot_ = 4.5 × 10^−4^ M and [HCO_3_^−^] _tot_ = 2.0 × 10^−3^ M, containing as well Na, K, Mg, Cl and SO_4_^2−^. Sorption experiments were conducted at S/L = 20 g L^−1^ with 9 different solid phases, including calcite with particle diameter size of 63–90 μm. No information was provided on the initial concentration of ^14^C. Differences in the absolute *R*_d_ values determined in this work and reported by Allard and co-workers could be eventually caused by differences in particle size of the materials used in both studies, and accordingly in surface area. Moreover, differences in *R*_d_ might be partially influenced by the different pH, [Ca]_tot_ and [HCO_3_^−^]_tot_ in both experimental datasets. Fig. 2a includes also the experimental results reported by Boylan and co-workers, who investigated the uptake of ^14^C by calcite at pH = 8.87, [Ca]_tot_ = 5 × 10^−5^ M and *C*_tot_ = 2.5 × 10^−3^ M within *t* = 7–30 days.^11^*R*_d_ values reported by Boylan *et al.*^11^ are lower than those reported in this work, but in line with data reported by Allard *et al.*^10^ Besides the differences in terms of pH, [Ca] and C_tot_, we note that the surface area of the calcite used by Boylan *et al.*^11^ (0.289 m^2^ g^−1^) was significantly lower than the calcite material used in this work (0.56 m^2^ g^−1^). Such differences could well explain the discrepancies observed between *R*_d_ values determined in this work and calculated from data reported in Boylan *et al.*^11^

Fig. 2b shows sorption data for the uptake of ^14^C by calcite as a function of the S/L ratio. The two horizontal solid lines represent the lower (0.1 L kg^−1^) and upper (10^2^ L kg^−1^) limits proposed by Ochs *et al.*^2^ for the uptake of ^14^C by cement in the degradation stage IV, *i.e.*, calcite. These upper and lower boundaries are indeed based on the sorption data reported by Allard and co-workers.^10^ The figure shows a slight decrease of *R*_d_ values with increasing S/L ratio at short contact times, which are expectedly dominated by adsorption processes. Similar observations have been previously described for sorption processes involving other solid phases, *e.g.*, cement or granite.^2,31,32^ However, there is no definitive explanation for this observation so far.

**Fig. 3:**
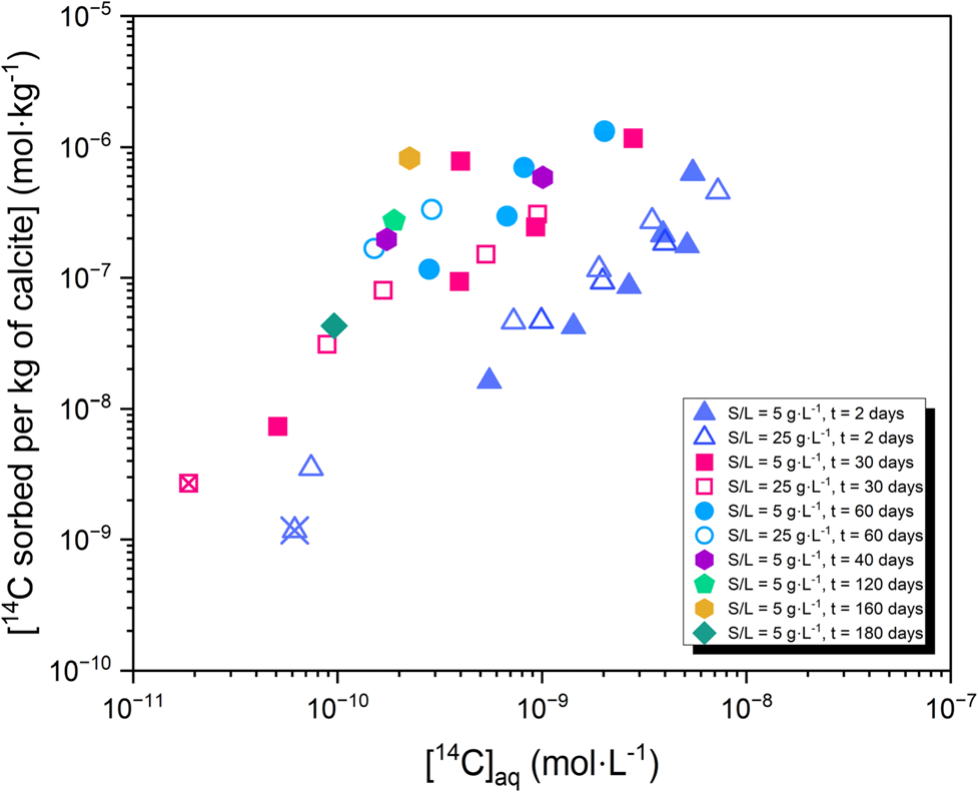
Sorption isotherms for the uptake of ^14^C by calcite at S/L = 5 g L^−1^ (full symbols) and 25 g L^−1^ (empty symbols). Batch sorption experiments were conducted for equilibration times of *t* = 2–180 days. Crossed symbols are at or below the detection limit.

Sorption isotherms in Fig. 3 show a linear trend over two orders of magnitude in terms of ^14^C aqueous concentration ([^14^C]_aq_). As observed in the sorption kinetics study (see Fig. 2), an increase of the uptake is observed with time, shifting the corresponding isotherms towards lower aqueous and higher sorbed ^14^C.

As described in the literature, calcite recrystallization is a key driving force for the incorporation of ^14^C *via* isotopic exchange.^9,33^ Heberling and co-authors^9^ reported that the recrystallization is a surface free-energy driven process and proposed a direct dependency on particle roughness, particle size and morphology. An increase of roughness (higher surface amorphization) and decrease of particle size contribute to an increase of surface free energy and, consequently, of recrystallization rates.^9^ Moreover, it is important to consider that calcite particles minimize their surface free energy during recrystallization, accordingly resulting in a decrease of the recrystallization rate during the reaction, which may affect the rate of ^14^C incorporation.^34^

#### 3.2.2 Modelling and mechanistic understanding of calcite recrystallization

**Fig. 4:**
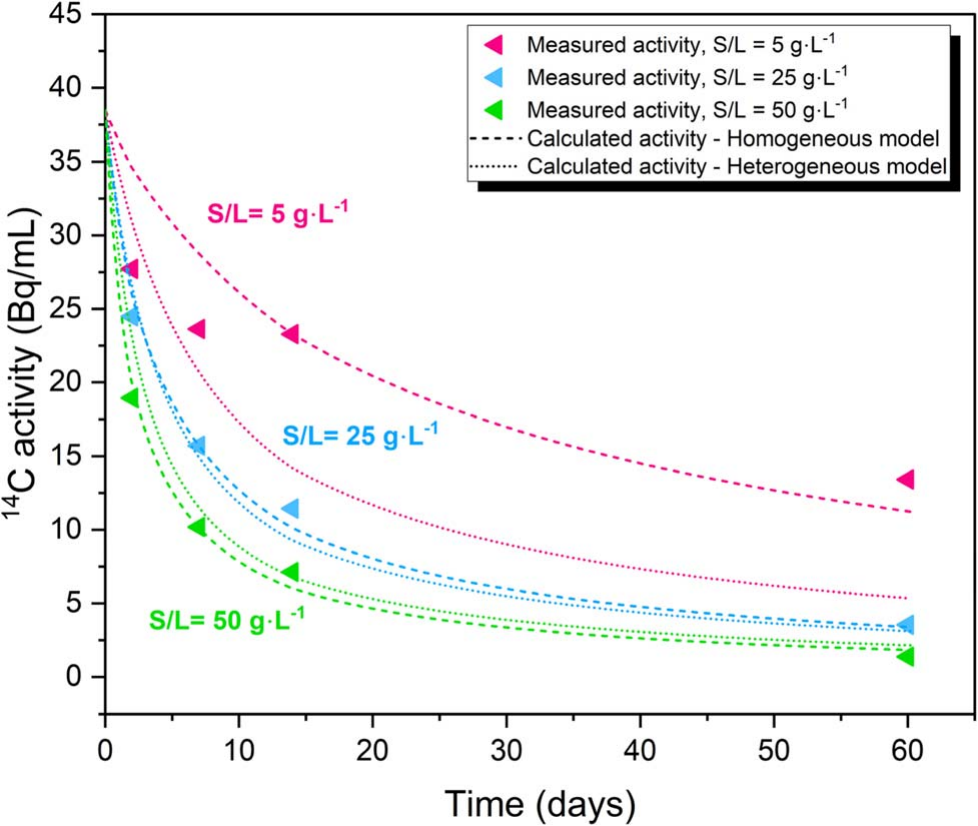
Comparison of experimental and calculated ^14^C activity as a function of time. Calculations conducted with the homogeneous (dashed line) and heterogeneous (dotted line) models, considering the average recrystallization rates summarized in Table 3.

The fraction of recrystallized and the recrystallization rates for the ^14^C-calcite system determined on the basis of the homogeneous and heterogeneous models are shown in Fig. S8 and S9 (SI), respectively. The fraction of recrystallized calcite (% Cc) increases as a function of the contact time (Fig. S8, SI), with greater fractions of recrystallized calcite being estimated by the homogeneous model as a consequence of the model assumptions (see Section 2.6). Fig. 4 shows the experimentally measured ^14^C activity as a function of time, compared with model calculations (homogeneous and heterogeneous models) conducted using the recrystallization rates summarized in Table 3. As shown in Table 3, average recrystallization rates quantified with the homogenous model results in *r*_recr_ = (1.4 ± 0.2)·10^−10^ mol m^−2^ s^−1^ and a fraction of recrystallized calcite of (3 ± 0.5)%, which corresponds to (100 ± 10) calcite monolayers reacted within 60 days. Less consistent rates are determined from the heterogeneous model, with *r*_recr_ = (2 ± 2)·10^−10^ mol m^−2^ s^−1^, corresponding to ≈ 90 calcite monolayers. These recrystallization rates are in agreement with the rates reported previously by Heberling and co-workers for a coarse calcite material with surface area of ≈ 0.2–0.6 m^2^ g^−1^.^9^

**Table 3:** Recrystallization rates (*r*_recr_) obtained from the fitting of both heterogeneous and homogeneous model for the ^14^C-calcite system with S/L = 5, 25 and 50 g L^−1^. Uncertainties for the recrystallization rates are reported with a 95% of confidence interval, while uncertainties of the average values calculated as two times the standard deviation

S/L, [g L^−1^]	*r*_recr_, [mol m^−2^ s^−1^] homogeneous model	*r*_recr_, [mol m^−2^ s^−1^] heterogeneous model
5	(1.61 ± 1.10)·10^−10^	(3.48 ± 2.40)·10^−10^
25	(1.38 ± 0.28)·10^−10^	(1.28 ± 0.39)·10^−10^
50	(1.33 ± 0.38)·10^−10^	(9.50 ± 3.15)·10^−11^
Average	(1.4 ± 0.2)·10^−10^	(2 ± 2)·10^−10^

**Fig. 5:**
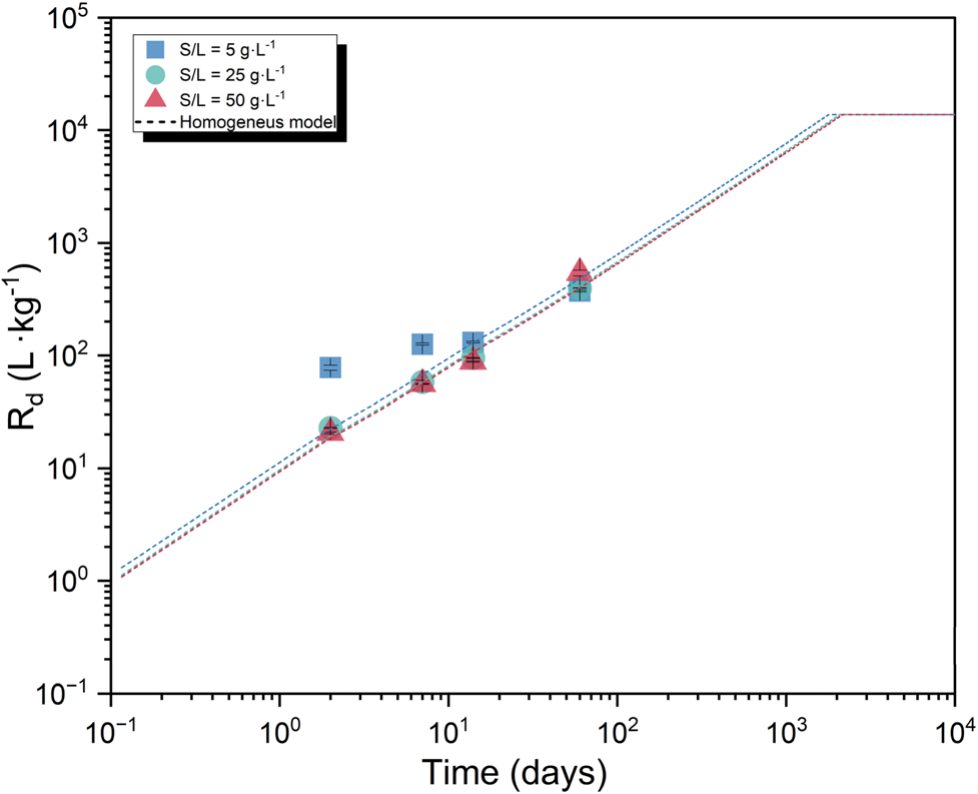
Time evolution estimated for the uptake of ^14^C by calcite according with the homogeneous recrystallization model derived in this work and considering the recrystallization rates reported in Table 3.

Average recrystallization rates estimated according to the homogenous model allow the prediction of *R*_d_ values for the uptake of ^14^C by calcite in the long-term. As shown in Fig. 5 the thermodynamic equilibrium implying the full recrystallization of calcite would result in *R*_d_ ≈ 10^4^ L kg^−1^, *i.e.* more than one order of magnitude greater than the highest value determined in this work, and more than two orders of magnitude greater than the upper limit reported by Ochs *et al.*^2^ Nevertheless, the assumption of thermodynamic equilibrium leading to complete recrystallization of calcite must be considered with some precaution. Calcite recrystallization is understood as a process minimizing the surface-free energy: with the progress of the reaction, the driving force decreases and consequently also the recrystallization rates decrease.^9^ Moreover, passivation and the presence of impurity ions may represent a further limit to calcite bulk recrystallization. Hence, the distribution ratio estimated in this work assuming complete equilibration, *R*_d_ ≈ 10^4^ L kg^−1^, should be considered only as an upper limit for the retention of ^14^C by calcite.

### 3.3 Impact of ISA and NaCl on ^14^C retention

#### 3.3.1 Uptake of ^14^C in the presence of ISA

The chemical stability of ISA in the weakly alkaline conditions defined by the equilibrium with calcite under air was investigated before initiating the sorption experiments with ^14^C. The ^1^H NMR spectra collected (see Fig. S10, SI) shows the typical ^1^H signals of ISA in open chain conformation and are well in agreement with the NMR analyses reported in Brinkmann *et al.* and Jo *et al.*^35,36^ No evident changes in the proton signals (new features, shift in peak position or changes in peak shape) have been identified within the investigated time frame, *t* = 2–160 days. These results confirm the stability of ISA under the considered boundary conditions, at least for *t* ≤ 160 days.

**Fig. 6:**
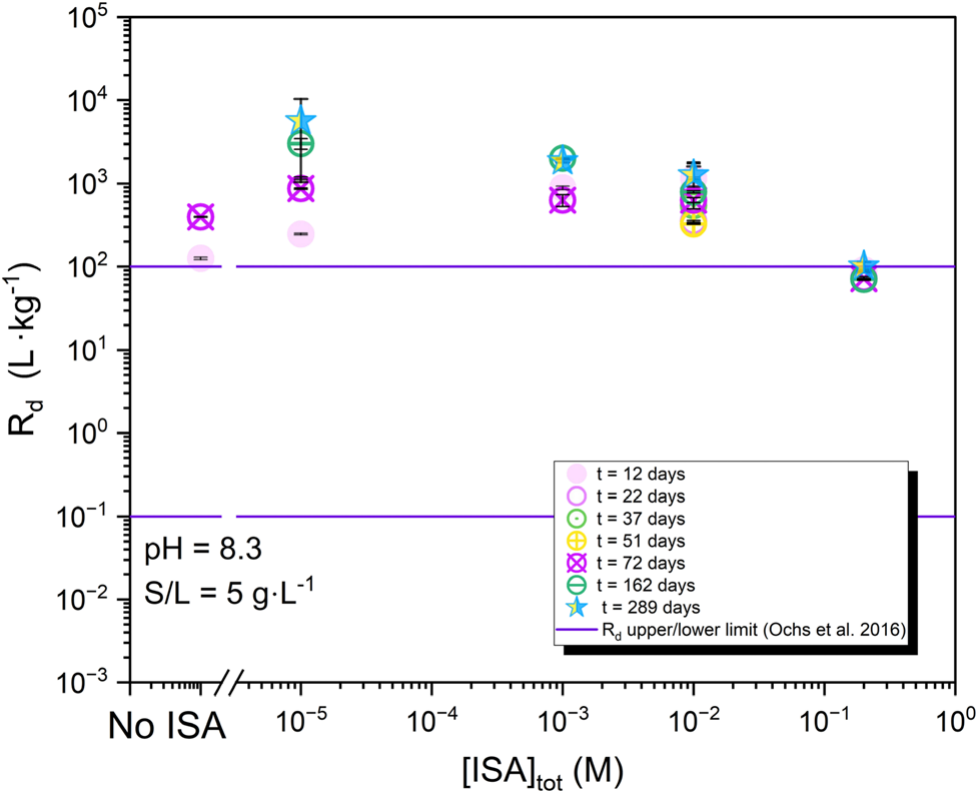
Distribution ratios (*R*_d_) determined for the uptake of ^14^C on calcite in presence of ISA ([ISA]_tot_ = 10^−5^–0.2 M, S/L = 5 g L^−1^). Sorption batch experiments were monitored for an equilibration time of 12 ≤ *t* (days) ≤ 289. Solid lines represent the upper and lower *R*_d_ limits reported by Ochs *et al.*^2^

Fig. 6 shows the distribution ratios determined for the uptake of ^14^C by calcite in the presence of ISA (10^−5^ M ≤ [ISA]_tot_ ≤ 0.2 M), S/L = 5 g L^−1^ and *t* = 12–289 days. Similar results were obtained for S/L = 25 g L^−1^ (see Fig. S11 and S12, in the SI). In general ISA has a relatively minor impact on the uptake of ^14^C by calcite. At low ligand concentrations, ^14^C retention increases slowly with increasing contacting time, confirming the results obtained for the ISA-free system (see Section 3.2.1). However, with increasing ligand concentration the temporal evolution of the *R*_d_ values decreases, virtually remaining constant for up to 289 days at the highest ISA concentration (0.2 M). This observation possibly reflects that although adsorption of ^14^C is not affected by ISA, the presence of this organic ligand may (partially) inhibit calcite recrystallization, subsequently altering the incorporation of ^14^C into calcite with time. Indeed, the impact of the adsorption of organic ligands on calcite recrystallization is extensively discussed in the literature for ligands containing different functional groups,^37,38^ long-chain carboxylic acids^39^ polysaccharides^40^*etc.* Brune reported also that glucose and sucrose adsorbates delayed the onset and progression of crystallization of amorphous calcium carbonate.^41^

**Fig. 7:**
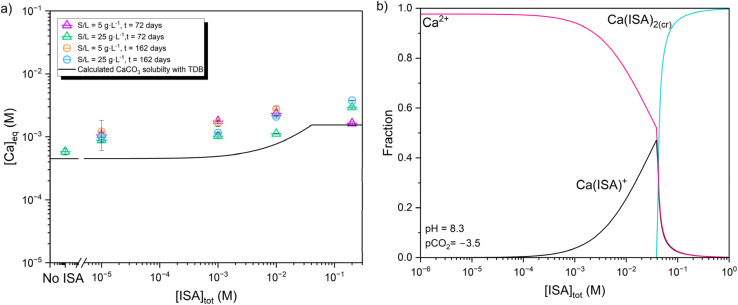
(a) Experimentally measured Ca concentrations in calcite systems under increasing ISA concentration (10^−5^ M ≤ [ISA]_tot_ ≤ 0.2 M) at contact times *t* = 72 and 162 days. Solid line corresponds to the solubility of calcite as calculated with the Thermochimie database at pH = 8.3 and pCO_2_ = −3.5.^26^ (b) Ca speciation underlying the solubility curve of calcite shown in Fig. 7a. Calculations conducted with the ThermoChimie database v.12a.^26^

The presence of ISA in solution triggers also the increase of the total Ca concentration in the aqueous phase (Fig. 7a). This increase is consistent with the formation of weak complexes, *i.e.* Ca(ISA)^+^, although the precipitation of Ca(ISA)_2_(cr) is also predicted at [ISA]_tot_ > 0.1 M according to thermodynamic calculations using the ThermoChimie database v.12a (Fig. 7b). The slight apparent decrease of the *R*_d_ values of ^14^C observed at [ISA]_tot_ = 0.2 M could be related to the slight dissolution of calcite, although other effects like the impact of ISA on the surface properties of calcite could also play a role.

#### 3.3.2 Uptake of ^14^C in the presence of NaCl and ISA + NaCl

**Fig. 8:**
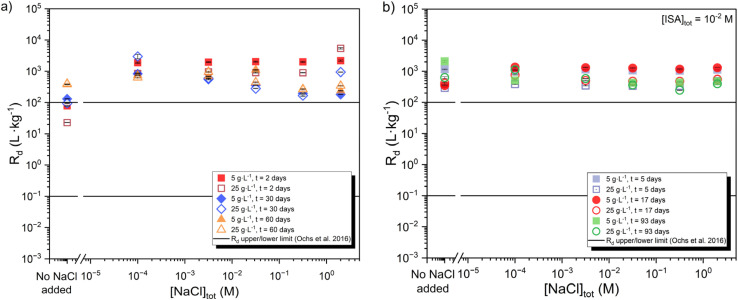
Distribution ratios (*R*_d_) determined for the uptake of ^14^C by calcite at pH_m_ ≈ 8.3 (a) in the absence and (b) in the presence of ISA ([ISA]_tot_ = 10^−2^M) as a function of increasing chloride concentration ([NaCl]_tot_ = 10^−5^–2 M) and S/L = 5 and 25 g L^−1^. ^14^C uptake was monitored for contact times of *t* = 2–60 and 5–93 days in the absence and presence of ISA, respectively. Solid lines represent the upper and lower *R*_d_ limits reported by Ochs *et al.* for the uptake of ^14^C by calcite.^2^

Fig. 8 shows the uptake of ^14^C in presence of chloride (10^−4^ M ≤ [NaCl]_tot_ ≤ 2 M) at contact times of 2 to 60 days, both in the absence (Fig. 8a) and presence (Fig. 8b) of [ISA]_tot_ = 0.01 M. For both systems, *R*_d_ values determined for the uptake of ^14^C in the presence of enhanced chloride concentrations are in line but slightly higher than those determined in chloride-free systems. Most of the calculated *R*_d_ values are found slightly above the upper limit reported by Ochs *et al.*^2^ On the other hand, the ^14^C uptake in chloride containing systems does not follow the same increasing trend with time observed for chloride-free system (see Section 3.2). This may hint towards the impact of chloride on the surface properties and recrystallization of calcite, as discussed above for calcite systems containing ISA. Note also that Quian and co-workers reported a significant decrease of the crystallization rates of calcite at high chloride concentrations, *i.e.*, 3.5 to 14 wt%,^42^ in line with the observations obtained in this work.

The surface electrokinetic properties of calcite are known to be impacted by the presence of Na^+^ and Cl^−^ ions in solution. Based on a series of streaming potential and electrical conductivity measurements, Li and co-workers developed a new approach for the characterization of the electrokinetic properties of calcite in contact with increasing NaCl concentrations (1 × 10^−3^ M ≤ [NaCl]_tot_ ≤ 5 × 10^−2^ M) and within the pH range 5.5 ≤ pH ≤ 10.5.^43^ At pH ≈ 8.3, the authors reported zeta potentials increasing from ≈ −40 mV to ≈ −20 mV at [NaCl] = 1 × 10^−3^ to 5 × 10^−2^ M, respectively. This trend was also confirmed by Al Mahrouqi and co-workers, who investigated the evolution of the zeta potentials of three natural calcite samples with increasing NaCl concentrations (4 × 10^−2^ M < [NaCl]_tot_ < 5 M).^44^ In calcite systems containing 2 M NaCl concentrations, the authors reported zeta potentials ranging from ≈ −6 mV (Portland calcite) to ≈ +1 mV (Estaillades calcite). Al Mahrouqi and co-workers observed an increase of the Ca concentration in solution with increasing NaCl concentration. This was explained by a decreased interaction of Ca^2+^ ions with the calcite surface due to the increasing occupancy of hydrated Na^+^ ions in the diffuse layer. We note that the recent surface complexation model for calcite–water interface in the presence of NaCl derived by Heberling and co-workers suggest that the outer-sphere sorption of ^14^C plays a minor role in the uptake in chloride reach systems.^45^

**Fig. 9:**
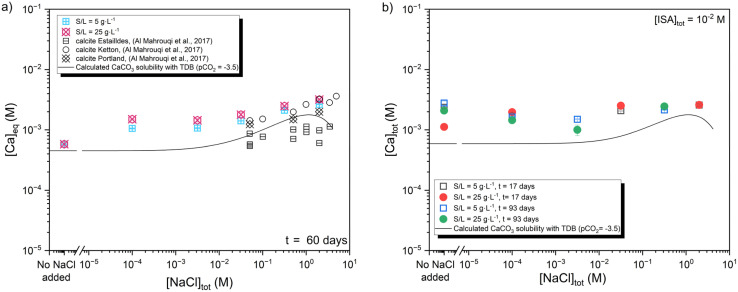
Experimentally measured Ca concentrations in calcite systems under increasing NaCl concentrations (10^−4^ M ≤ [NaCl]_tot_ ≤ 2 M), pH_m_ ≈ 8.3, S/L = 5 and 25 g L^−1^ (a) in the absence of ISA at a contact time of *t* = 60 days, and (b) in the presence of ISA ([ISA]_tot_ = 10^−2^M) at contact times of *t* = 17 and 93 days. Solid lines in Figures a and b correspond to the solubility of calcite calculated using the Thermochimie database v.12a and SIT for ionic strength corrections.^26^

In systems containing ISA and chloride, the presence of this organic ligand is expected to further impact the surface properties of calcite, as discussed in Section 3.2.2 for chloride-free systems and reported in the literature for several organic ligands containing –COOH and –OH functional groups. The presence of NaCl and/or ISA can possibly trigger the dissolution of the most reactive sites/particles prone to dissolution-precipitation processes, but the overall impact quantified in terms of [Ca] evolution as a function of NaCl or [ISA] is relatively minor, as shown in Fig. 7 (ISA system) and Fig. 9 (Cl and ISA + Cl systems).

A slight increase in the total Ca concentration with increasing NaCl concentrations is observed for both S/L = 5 and 25 g L^−1^ systems (Fig. 9a). These results are in line with the evolution of the Ca concentration reported by Al Mahrouqi *et al.* for three natural calcite samples under increasing NaCl concentrations (Fig. 9a).^44^ From a macroscopic perspective, the increase of Ca concentration is also consistent with the expected increase of calcite solubility, triggered by ion interaction processes in the aqueous phase, as calculated using the ThermoChimie database and SIT for ionic strength corrections (solid line in Fig. 9a). Note however that the fraction of the initially dissolved calcite in 2 M NaCl is very limited, *i.e.*, ≈ 6% and ≈ 1.2% at S/L = 5 and 25 g L^−1^, respectively. The presence of [ISA]_tot_ = 0.01 M results in a slight increase of Ca concentration compared to ISA-free system and independently of NaCl concentration (Fig. 9b). These observations are in line with the results obtained in the presence of ISA but absence of chloride. The solubility of calcite calculated for these boundary conditions using the ThermoChimie database and SIT for ionic strength corrections explains moderately well the Ca concentrations experimentally measured for this system.

## 4 Conclusions

The uptake of ^14^C by calcite was investigated in the presence and absence of ISA and chloride with a comprehensive series of batch sorption experiments at pH ≈ 8.3 under ambient atmosphere (air). Retention was investigated under the variation of [^14^C], S/L ratio, [ISA], [NaCl] over a timeframe of 2 ≤ *t* [days] ≤ 289. Calcite recrystallization was evaluated with dedicated series of inactive batch experiments, involving the characterization of calcite as a function of time in terms of crystalline structure, surface morphology and BET surface area.

The crystalline structure of calcite in the absence and presence of chloride ([NaCl] = 2 M) remains mostly unaffected, whereas the evolution of the surface morphology highlights surface dissolution and precipitation processes. BET surface area decreases with time, thus supporting the increase of the average particle size through recrystallization phenomena.

Sorption kinetics experiments show a moderate uptake of ^14^C at short contact times (*R*_d_ ≈ 20–70 L kg^−1^ at *t* = 2 days), followed by a systematic increase of the uptake with time resulting in *R*_d_ ≈ 10^3^ L kg^−1^ at *t* = 60 days. In line with the literature,^2^ these results are interpreted in terms of fast surface adsorption followed by the slower incorporation of ^14^C into the calcite structure through recrystallization phenomena. Sorption isotherms confirm the linear sorption behavior over ≈ 10^−11^ M ≤ [^14^C] ≤ ≈ 10^−8^ M.

To explore the role of calcite recrystallization in the retention of ^14^C, results obtained in the sorption kinetics experiments in combination with BET measurements of the surface area were interpreted and compared in terms of homogeneous and heterogeneous recrystallization models. The homogeneous model best describes the experimental observations obtained in this work, with average recrystallization rates resulting in *r*_recr_ = (1.4 ± 0.2)·10^−10^ mol m^−2^ s^−1^. These values are in line with recrystallization rates previously reported in the literature for the uptake of ^45^Ca by calcite.^9^ The homogeneous recrystallization model was accordingly used for the prediction of ^14^C uptake in the long-term. Such predictions assume constant rates and complete calcite recrystallization, and thus may only be considered as upper limits of ^14^C sequestration in view of possible secondary processes inhibiting crystallization, *e.g.*, passivation.

ISA has a minor impact on the overall distribution ratios of ^14^C determined within 10^−5^ M ≤ [ISA] ≤ 0.1 M. The slight decrease in sorption observed at [ISA] = 0.2 M is possibly related to the dissolution of calcite through the formation of Ca(ISA)^+^ and Ca(ISA)_2_(cr), as evidenced by the quantification of Ca concentration in the aqueous phase and thermodynamic calculations. Remarkably, no increase of the ^14^C uptake with time is observed in the presence of ISA, which could be related to the alteration of the calcite recrystallization process triggered by the impact of ISA on the surface properties of calcite.

The presence of NaCl results in a slight increase of the ^14^C uptake. Ion interaction processes occurring at high NaCl concentrations result in the slight increase of calcite dissolution, as confirmed by the quantification of Ca concentration in the aqueous phase and thermodynamic calculations. As in the case of ISA, the presence of NaCl minimizes the increase of ^14^C uptake with time, eventually due to the alteration of the recrystallization process. Similar distribution coefficients are obtained in sorption experiments containing both ISA and NaCl.

This work provides a quantitative description and improved process understanding for the uptake of ^14^C by calcite under conditions relevant for an advanced cement degradation stage in the context of near-surface disposal of L/ILW-SL.

## Author contributions

Rosa Ester Guidone: conceptualization, investigation, methodology, validation, visualization, writing – original draft, writing – review & editing; Nils Huber: investigation, methodology, validation; Frank Heberling: conceptualization, methodology, writing – review & editing; Thomas Sittel: investigation, methodology, validation, writing – review & editing; Natalia Palina: investigation, methodology, validation, writing – review & editing; Florian Bocchese: supervision, conceptualization, writing – review & editing, Stéphane Brassinnes: supervision, conceptualization, writing – review & editing; Marcus Altmaier: supervision, conceptualization, project administrator, writing – review & editing; Xavier Gaona: supervision, resources, conceptualization, methodology, project administrator, writing – review & editing.

## Conflicts of interest

There are no conflicts to declare.

## Supplementary Material

RA-015-D5RA05547D-s001

## Data Availability

The data supporting this article have been included as part of the SI. Supplementary information is available. See DOI: https://doi.org/10.1039/d5ra05547d.
