# Supplementary material for: Uptake of H14CO3−/14CO32− by calcite: impact of ISA and chloride
Source: RSC Adv. 2025 Oct 15;15(46):38762–73. doi: 10.1039/d5ra05547d (PMC12525538; doi:10.1039/d5ra05547d)
Supplement: RA-015-D5RA05547D-s001 [file RA-015-D5RA05547D-s001.pdf]

Supplementary Information (SI) for RSC Advances.
This journal is © The Royal Society of Chemistry 2025

**Supplementary Information (SI)**

Submitted to *RSC Advances*

# Uptake of $H^{14}CO_3^-$ / $^{14}CO_3^{2-}$ by calcite: impact of ISA and chloride

Rosa Ester Guidone[1,*], Nils Huber[1], Frank Heberling[1], Thomas Sittel[1], Natalie Palina[1], Florian Bocchese[2], Stéphane Brassinnes[2], Marcus Altmaier[1], Xavier Gaona[1,*]

[1] Karlsruhe Institute of Technology (KIT), Institute for Nuclear Waste Disposal (INE), Hermann-von-Helmholtz-Platz 1, 76344 Eggenstein-Leopoldshafen, Germany
[2]ONDRAF/NIRAS, Belgian Agency for Radioactive Waste and Enriched Fissile Materials, Brussels (Belgium)

## 1 Determination of the absolute activity

To determine the absolute sample activity (DPM), the measured activity (CPM) has been corrected by the appropriate counting efficiency, as shown in (1):

$$DPM = \frac{CPM_{sample}}{Efficiency} \tag{1}$$

The counting efficiency is determined through quench curve. The latest consists of a measurement of a series of 10 standards ($^{14}C$ low level quenched, Perkin Elmer) with a constant absolute activity and in which the number of quenching increases from standard to standard. Since the DPM of each standard is known and the CPM is measured, the counting efficiency is determined as follows (2):

$$Efficiency = \frac{CPM_{standard}}{DPM_{\ standard}} \tag{2}$$

The quench curve, plotted against the quench indicating parameter t-SIE (transformed-spectral index of an external standard), is then fitted to the standard points. More details are reported in.[1]

## 2 Calcite characterization

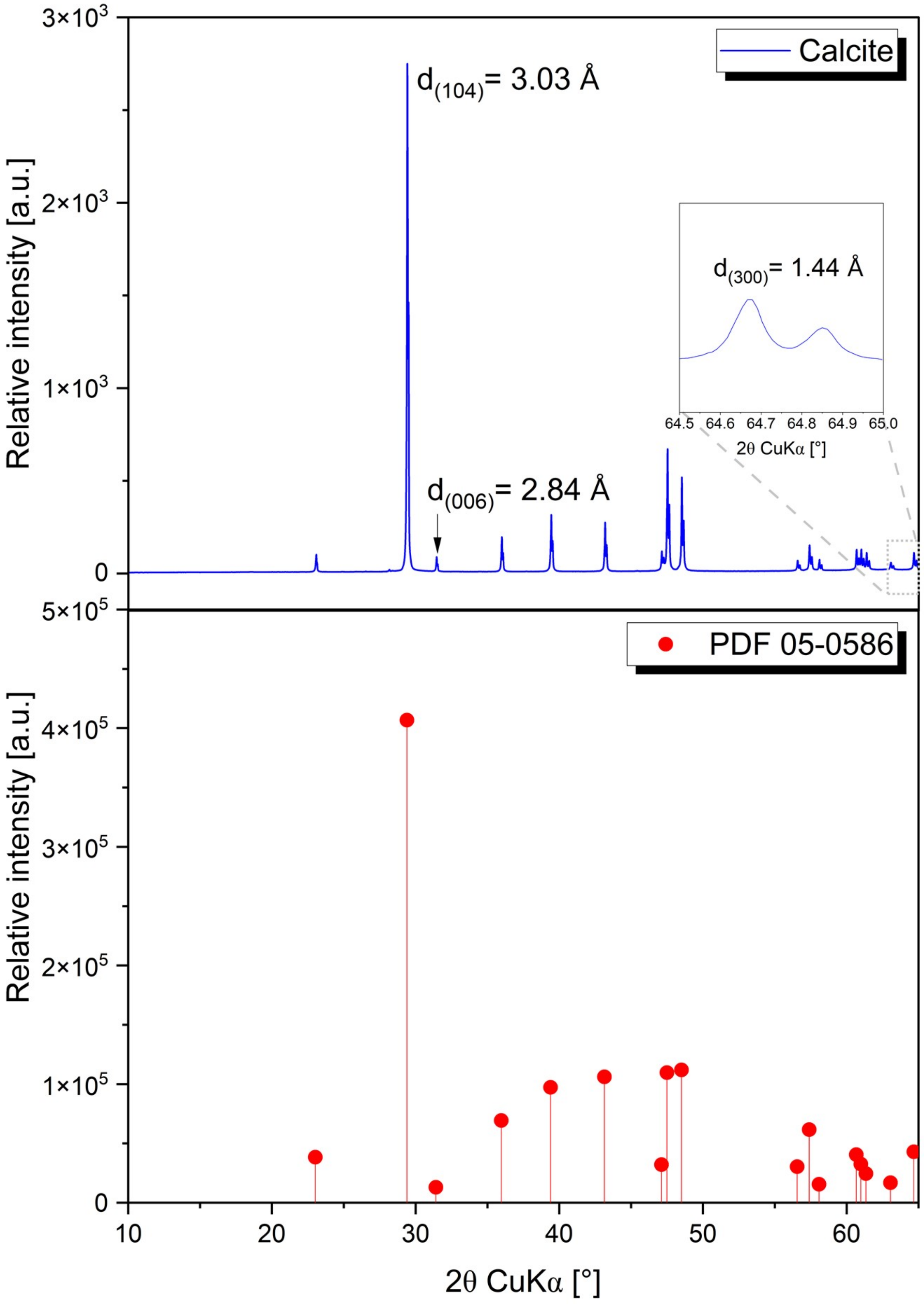


*Figure S1. Diffractogram obtained for the calcite material used for sorption and recrystallization experiments before equilibration. Bottom figure corresponds to the calcite reference diffractogram PDF 05-0586.*

## 3 Calcite recrystallization: chloride-free system

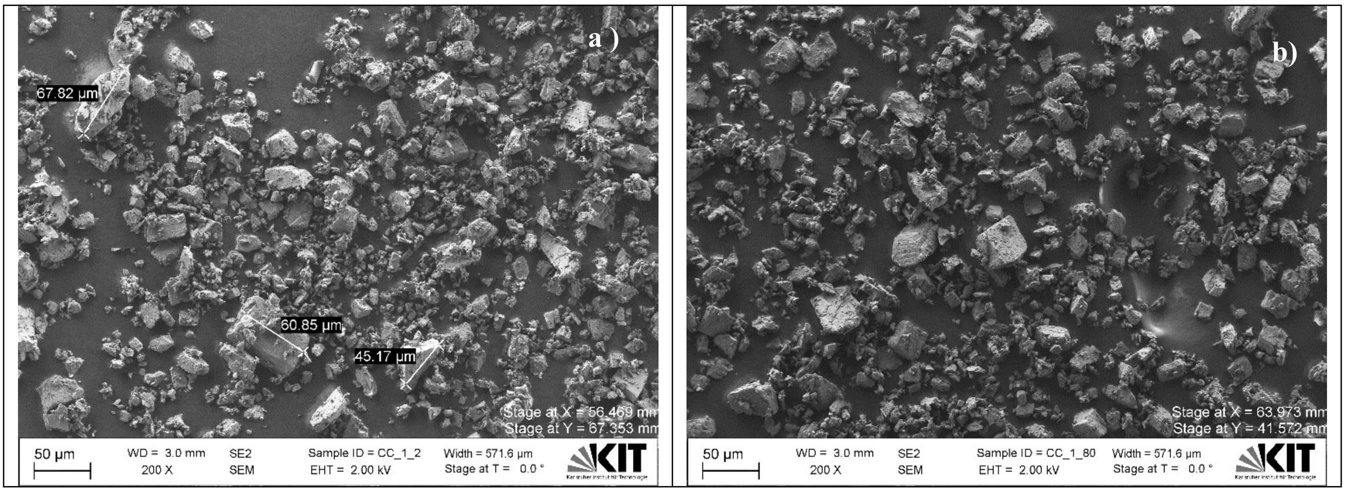


*Figure S2. Heterogeneity of calcite particles (a) after 2 days and (b) after 82 days of equilibration time. SEM images are reported with a magnification of X 200.*

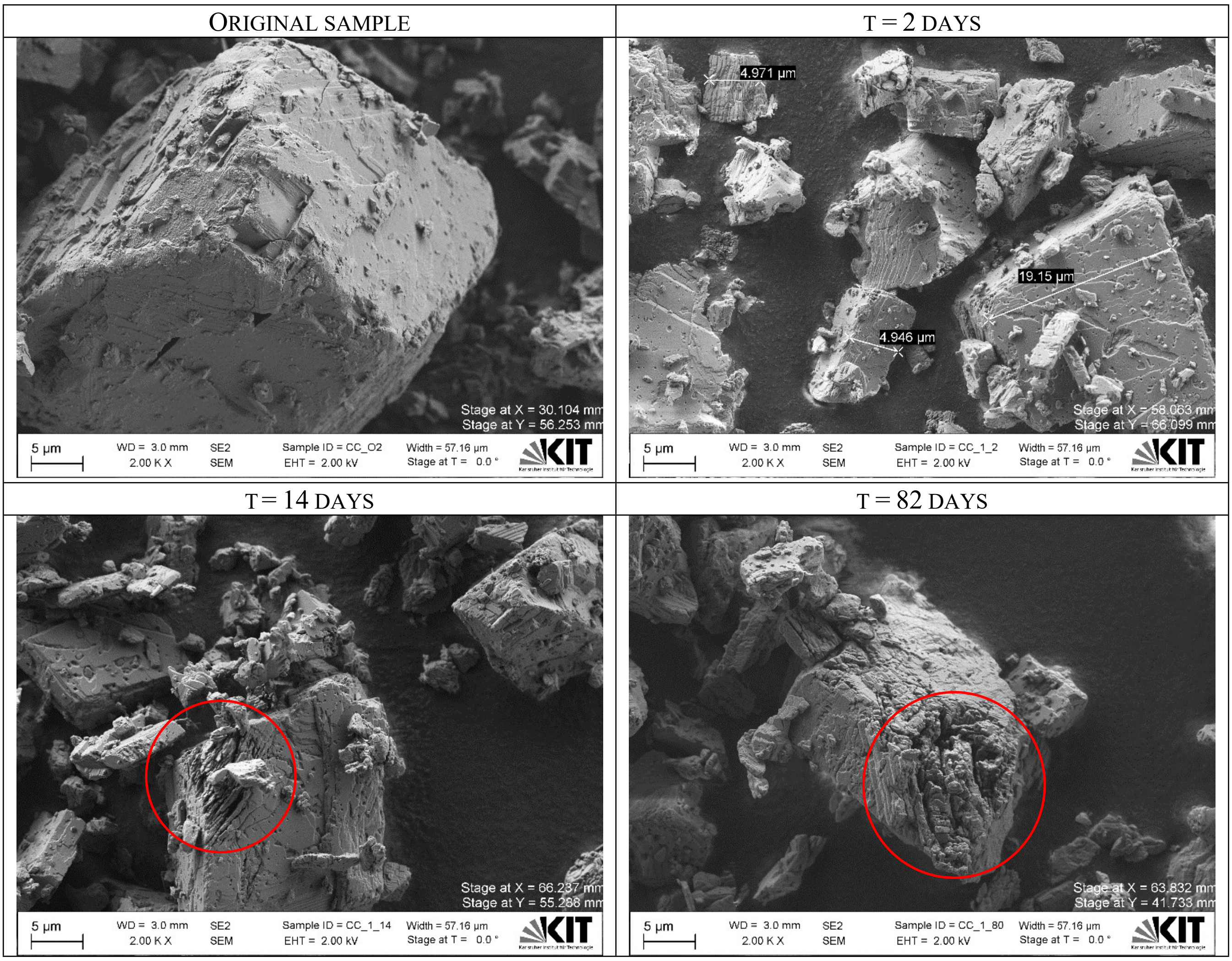


*Figure S3. Surface evolution of calcite particles in recrystallization experiments carried out for contacting times 2 < t (days) < 82. SEM images with a magnification of X 2000 are reported.*

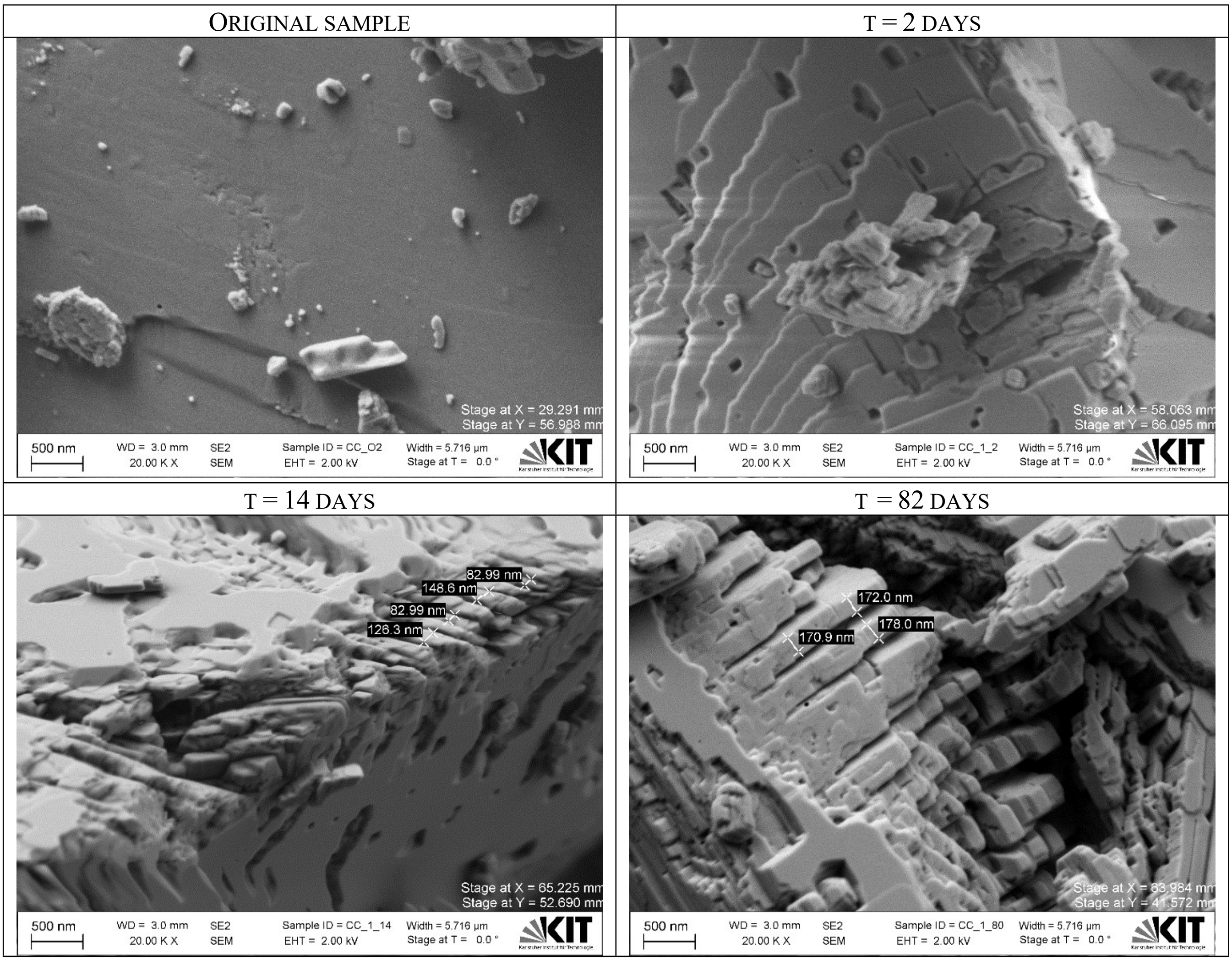


*Figure S4. Surface evolution of calcite particles in recrystallization experiments carried out for contacting times 2 < t (days) < 82. SEM images with a magnification of X 20000 are reported.*

# 4 Calcite recrystallization in the presence of chloride (NaCl)

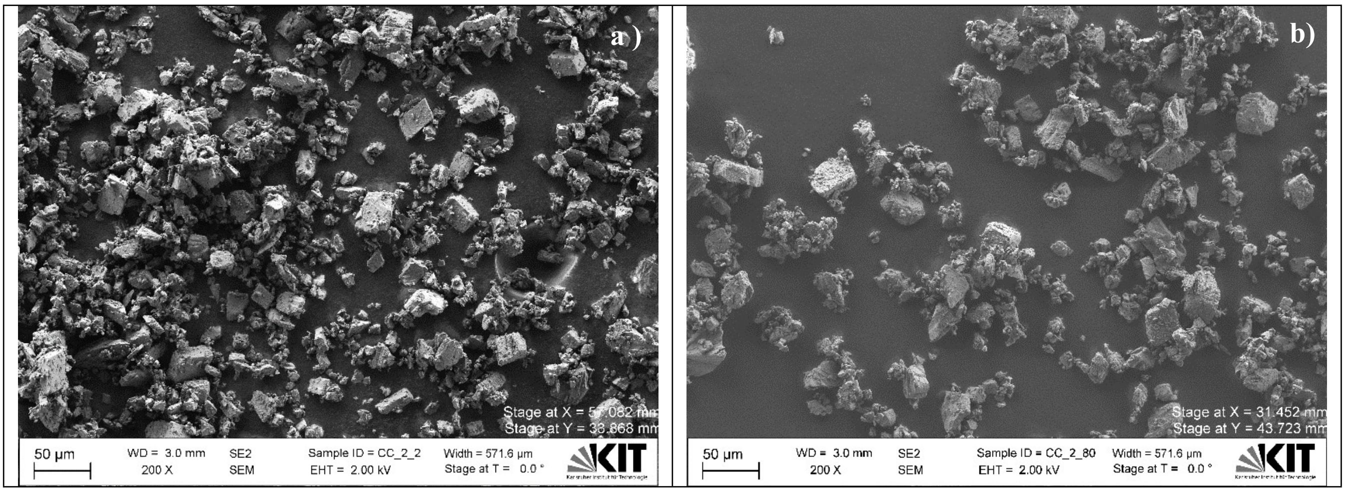


*Figure S5. Heterogeneity of calcite particles in presence of $[NaCl]_{tot}$= 2 M (a) after 2 and (b) after 82 days of equilibration. SEM images are reported with a magnification of X 200.*

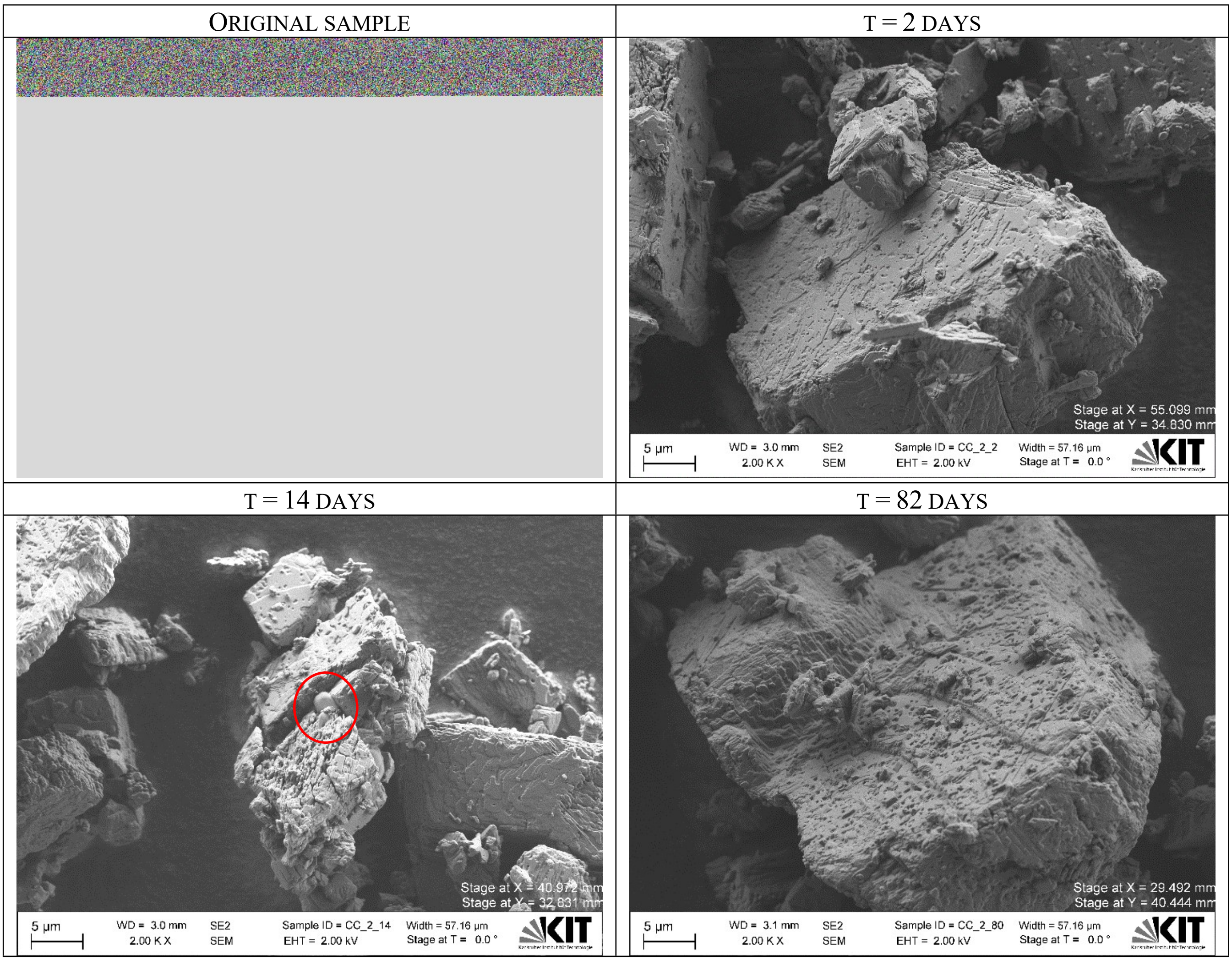


*Figure S6. Surface evolution of calcite particles in presence of NaCl in recrystallization experiments carried out for contacting times 2 < t (days) < 82 experiments and $[NaCl]_{tot}$= 2M. SEM images with a magnification of X 2000 are reported.*

| ORIGINAL SAMPLE | T = 2 DAYS |
|---|---|
| 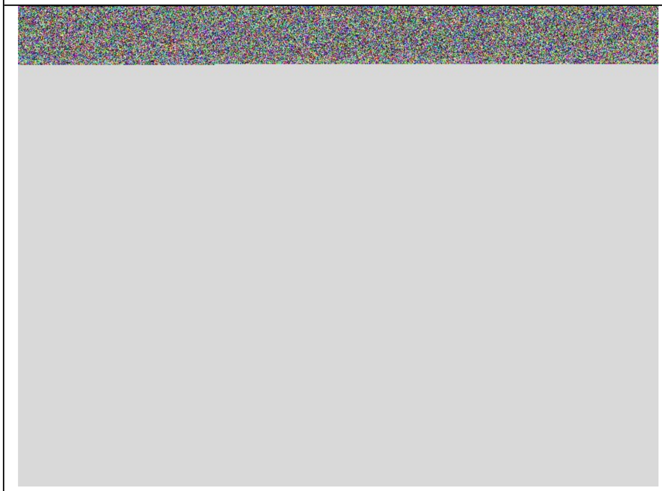 | 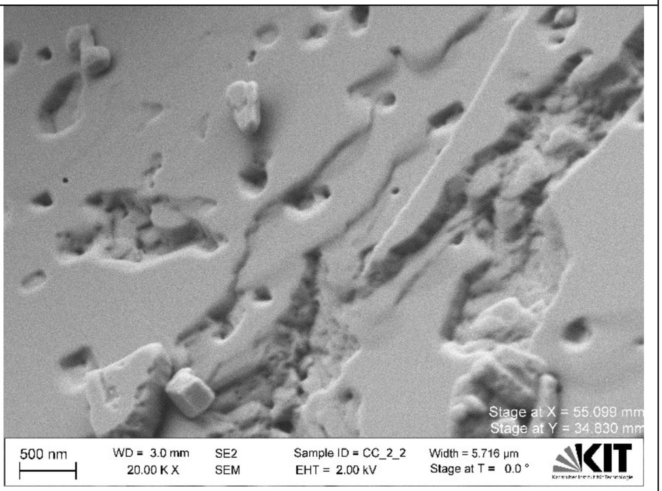 |
| T = 14 DAYS | T = 82 DAYS |
| 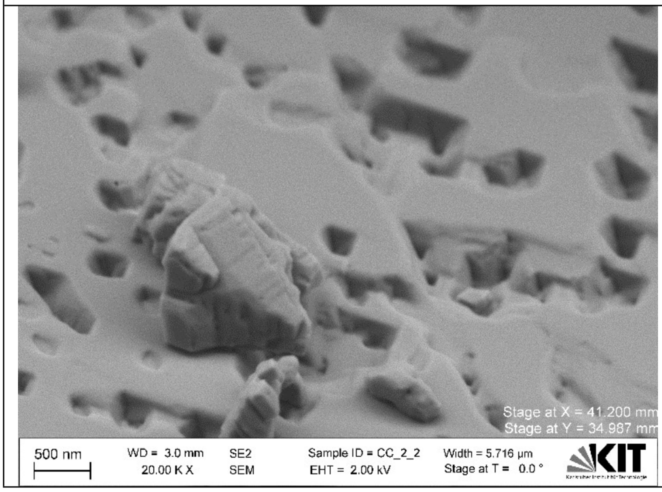 | 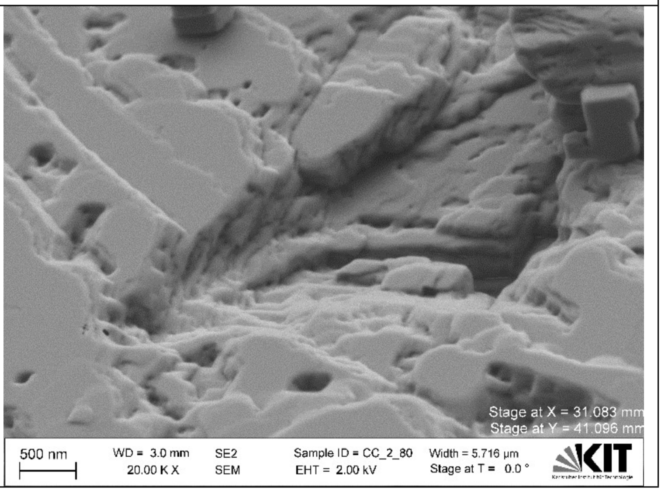 |

*Figure S7. Surface evolution of calcite particles in presence of NaCl in recrystallization experiments carried out for contacting times 2 < t (days) < 82 experiments and $[NaCl]_{tot}$= 2M. SEM images with a magnification of X 20000 are reported.*

## 5 Modelling and mechanistic understanding of calcite recrystallization

### 5.1 Calcite system

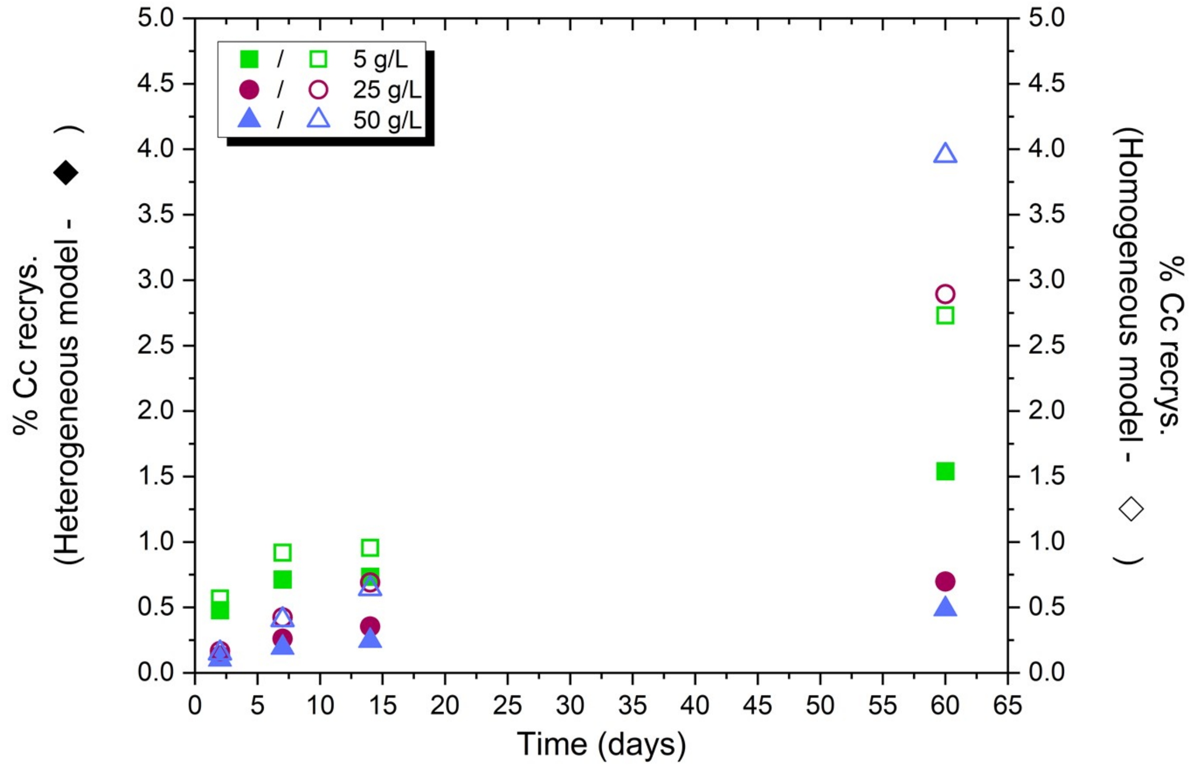


*Figure S8. Fraction of recrystallized calcite (%Cc) determined for the sorption series (Calcite-$^{14}C$) carried out with a S/L ratio = 5, 25 and 50 g·L$^{-1}$. Full and empty symbols represent the %Cc for the heterogeneous and the homogeneous model respectively.*

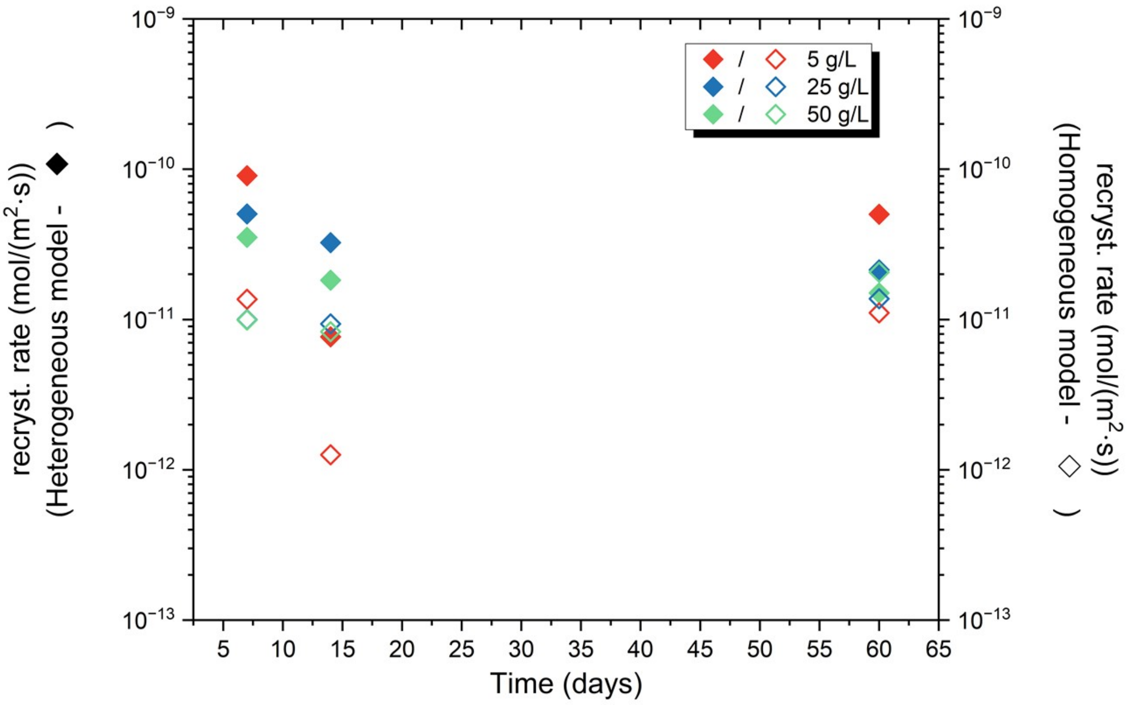


*Figure S9. Calcite recrystallization rates determined for the sorption series (Calcite-$^{14}C$) carried out with a S/L ratio = 5, 25 and 50 g·L$^{-1}$. Full and empty symbols represent the recrystallization rates for the heterogeneous and the homogeneous model respectively.*

## 6 Impact of ISA on $^{14}C$ retention by calcite

*Table S1. Equilibrium constants in the reference state considered in this work for thermodynamic calculations (speciation, solubility) of the Ca-ISA system. ISA stands for isosaccharinic acid.*

| Species | Log $K_0^S$ | Reaction | Reference |
|---|---|---|---|
| *Aqueous species* | | | |
| **$CaCO_3(aq)$** | 3.22±0.14 | $Ca^{2+} + CO_3^{2-} \leftrightarrow CaCO_3(aq)$ | ThermoChimie database [2] |
| **$CaHCO_3^+$** | 11.43±0.30 | $Ca^{2+} + H^+ + CO_3^{2-} \leftrightarrow CaHCO_3^+$ | ThermoChimie database [2] |
| **$CaOH^+$** | -12.78±0.30 | $Ca^{2+} + H_2O \leftrightarrow Ca(OH)^+ + H^+$ | ThermoChimie database [2] |
| **$HCO_3^-$** | 10.33±0.08 | $H^+ + CO_3^{2-} \leftrightarrow HCO_3^-$ | ThermoChimie database [2] |
| **$Ca(ISA)^+$** | 1.70±0.30 | $Ca^{2+} + ISA^- \leftrightarrow Ca(ISA)^+$ | ThermoChimie database [2] |
| **Ca(ISA)** | -10.4±0.50 | $Ca^{2+} + ISA^- \leftrightarrow Ca(ISA) + H^+$ | ThermoChimie database [2] |
| **ISA** | 4.00±0.50 | $H^+ + ISA^- \leftrightarrow ISA$ | ThermoChimie database [2] |
| *Solid species* | | | |
| **$Ca(ISA)_2(cr)$** | 6.40±0.20 | $Ca^{2+} + 2ISA^- \leftrightarrow Ca(ISA)_2(cr)$ | ThermoChimie database [2] |
| **Calcite** | 8.48±0.02 | $Ca^{2+} + CO_3^{2-} \leftrightarrow$ Calcite | ThermoChimie database [2] |
| **Portlandite** | -22.81±0.05 | $Ca^{2+} + 2H_2O \leftrightarrow$ Portlandite $+ 2H^+$ | ThermoChimie database [2] |

## 6.1 NMR characterization of ISA in calcite saturated pore water

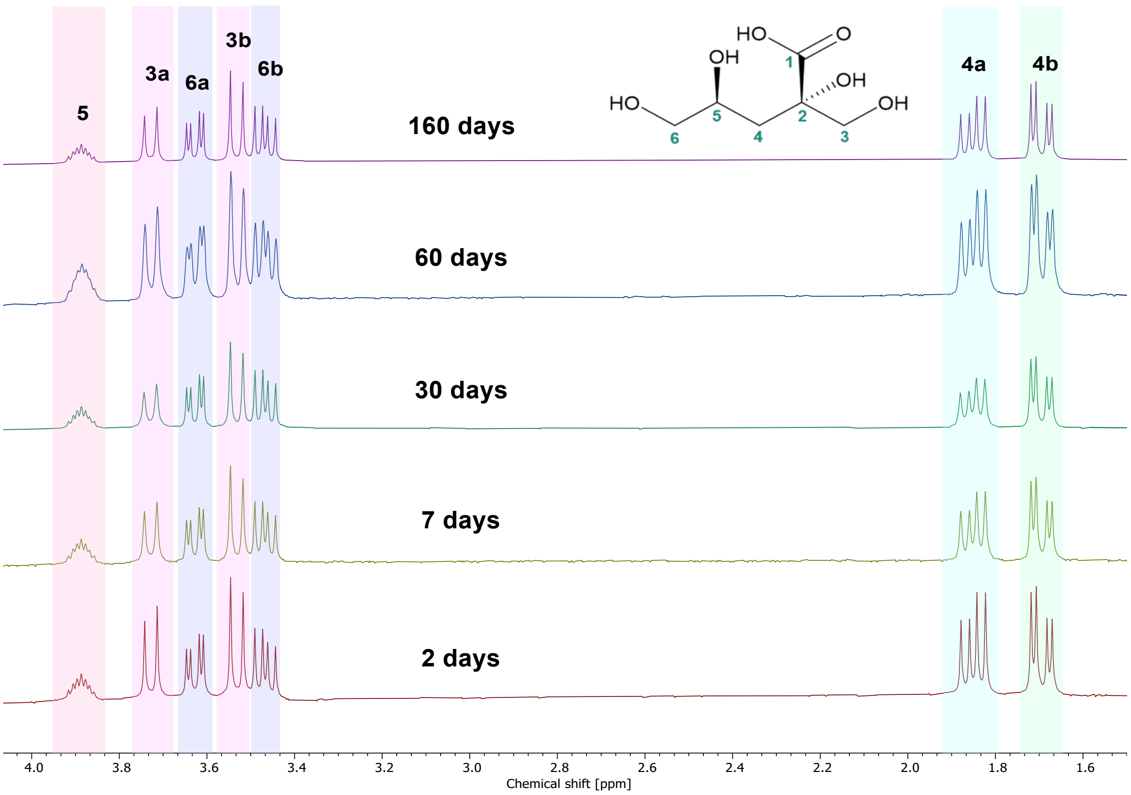


*Figure S10. $^{1}$H NMR spectra of ISA equilibrated in calcite pore water (pH=8.3) for equilibration times t = 2–160 days.*

### 6.2 $^{14}$C uptake in presence of α-ISA

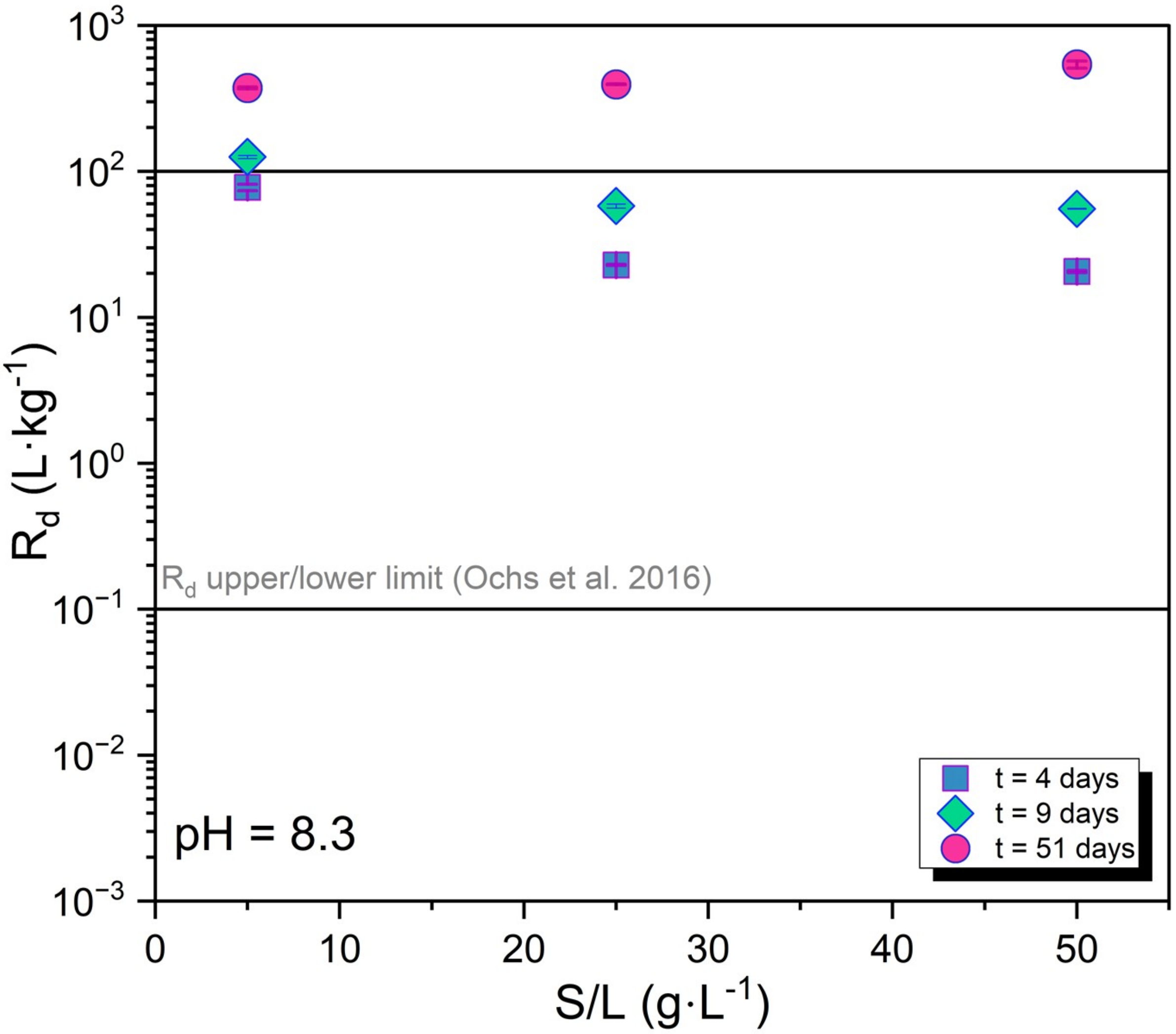


*Figure S11. Distribution ratios $R_d$ determined in the presence of $[ISA]_{tot}$ = $10^{-2}$ M for the uptake of $^{14}$C by calcite as a function of S/L ratio. Solid lines represent the upper and lower $R_d$ limits reported by Ochs et al.[3]*

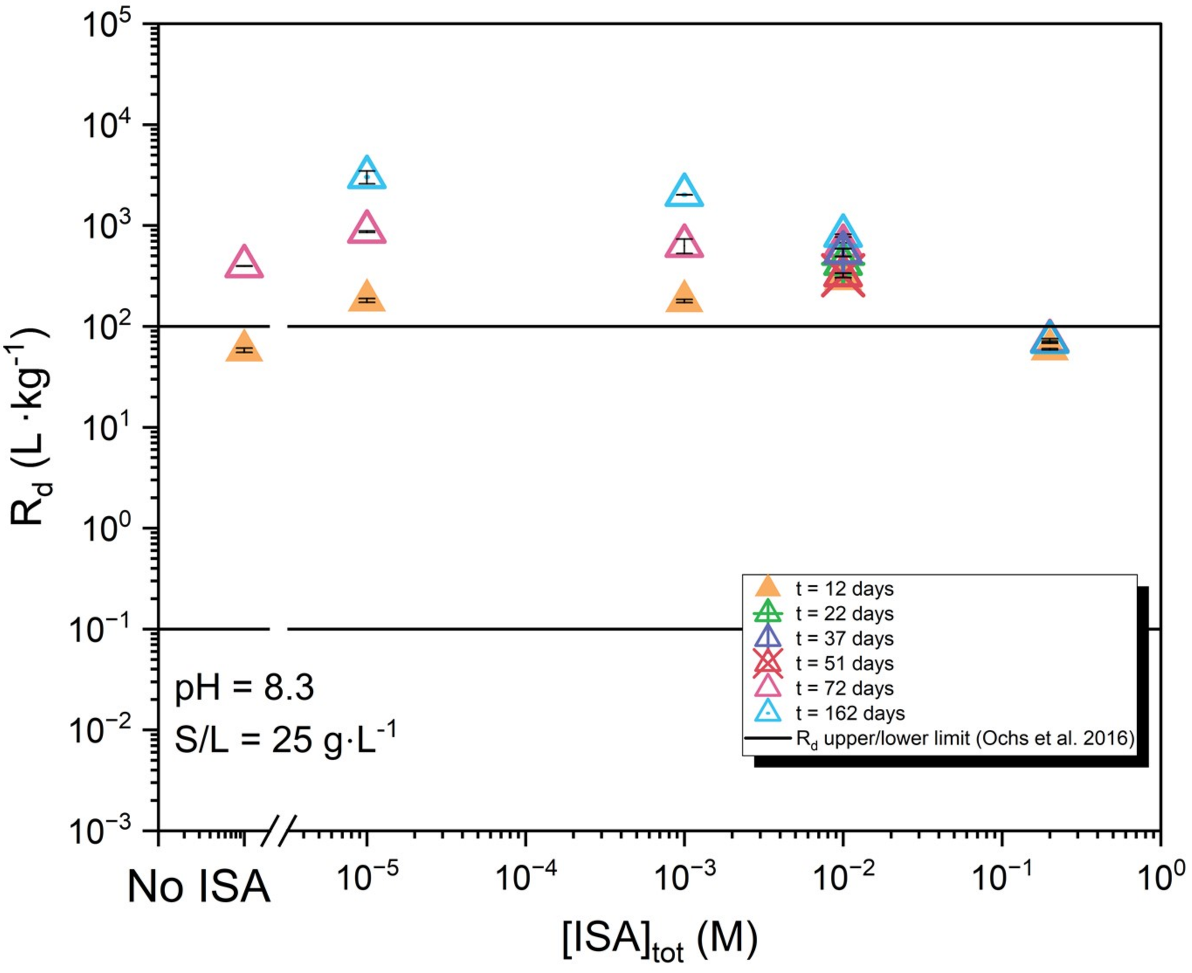


*Figure S12. Distribution ratios ($R_d$) determined for the uptake of $^{14}C$ by calcite in presence of ISA ($[ISA]_{tot}$ =$10^{-5}$–0.2 M, S/L = 25 g·$L^{-1}$ ). Sorption batch experiments monitored for contact times of 10 ≤ t (days) ≤ 160. Solid lines represent the upper and lower $R_d$ limits reported by Ochs et al.*[3]

## 7 Distribution coefficients determined in the sorption experiments

*Table S2. Distribution coefficients, $R_d$, determined in the sorption experiments for the $^{14}C$-calcite system.*

| Initial activity, ($Bq \cdot mL^{-1}$) | S/L, ($g \cdot L^{-1}$) | Time, (days) | $R_D$, ($L\ kg^{-1}$) | Error[A] | Experiment purpose |
|---|---|---|---|---|---|
| 38.5 | 5 | 2 | 77.7 | 3.9 | Sorption kinetics (variation of S/L ratio) |
| 38.5 | 5 | 7 | 125.7 | 2.9 | |
| 38.5 | 5 | 14 | 130.6 | 2.3 | |
| 38.5 | 5 | 60 | 373.5 | 5.9 | |
| 38.5 | 25 | 2 | 22.87 | 0.21 | |
| 38.5 | 25 | 7 | 57.9 | 1.9 | |
| 38.5 | 25 | 14 | 94.57 | 0.75 | |
| 38.5 | 25 | 60 | 395.7 | 2.2 | |
| 38.5 | 50 | 2 | 20.62 | 0.36 | |
| 38.5 | 50 | 7 | 55.53 | 0.35 | |
| 38.5 | 50 | 14 | 88.0 | 1.1 | |
| 38.5 | 50 | 60 | 541 | 31 | |
| 0.20 | 5 | 2 | 47 | 55 | Sorption isotherm (variation of $^{14}C$ initial activity) |
| 0.20 | 25 | 2 | 19.1 | 7.8 | |
| 2 | 5 | 2 | 63 | 12 | |
| 2 | 25 | 2 | 29.2 | 3.0 | |
| 5 | 5 | 2 | 60.9 | 1.0 | |
| 5 | 25 | 2 | 29.57 | 0.82 | |
| 10 | 5 | 2 | 78.2 | 1.4 | |
| 10 | 25 | 2 | 32.2 | 3.4 | |
| 20 | 5 | 2 | 62.9 | 4.9 | |
| 20 | 25 | 2 | 34.20 | 0.44 | |
| 0.20 | 5 | 30 | 144 | 91 | |
| 0.20 | 25 | 30 | [B] | - | |
| 2 | 5 | 30 | 238 | 37 | |
| 2 | 25 | 30 | 348 | 34 | |
| 5 | 5 | 30 | 263 | 10 | |
| 5 | 25 | 30 | 479 | 90 | |
| 10 | 5 | 30 | 1960 | 72 | |
| 10 | 25 | 30 | 284 | 12 | |
| 20 | 5 | 30 | 417 | 15 | |
| 20 | 25 | 30 | 322.5 | 5.0 | |
| 0.20 | 5 | 60 | [B] | - | |
| 0.20 | 25 | 60 | [B] | - | |

| | | | | | |
|---|---|---|---|---|---|
| 2 | 5 | 60 | 414 | 28 | |
| 2 | 25 | 60 | B | - | |
| 5 | 5 | 60 | 439 | 20 | |
| 5 | 25 | 60 | B | - | |
| 10 | 5 | 60 | 854 | 10 | |
| 10 | 25 | 60 | 1111 | 205 | |
| 20 | 5 | 60 | 652 | 17 | |
| 20 | 25 | 60 | 1154 | 54 | |
| 0.20 | 5 | 160 | B | - | |
| 0.20 | 25 | 160 | B | - | |
| 2 | 5 | 160 | B | - | |
| 2 | 25 | 160 | B | - | |
| 5 | 5 | 160 | B | - | |
| 5 | 25 | 160 | B | - | |
| 10 | 5 | 160 | 3642 | 523 | |
| 10 | 25 | 160 | B | - | |
| 20 | 5 | 160 | B | - | |
| 20 | 25 | 160 | 106192 | 34630 | |
| 5 | 5 | 40 | 2073 | 163 | |
| 20 | 5 | 40 | 1180 | 153 | |
| 20 | 5 | 160 | 2584 | 102 | |

[a] Uncertainties are reported as two times the standard deviation (2σ) of mean values.
[b] Measured value below the detection limit.

*Table S3. Distribution coefficients, $R_d$, determined in the sorption experiments for the calcite-$^{14}$C-ISA system carried out with $[ISA]_{tot}$ = $10^{-5}$-0.2 M.*

| Initial activity, (Bq·mL$^{-1}$) | S/L, (g·L$^{-1}$) | Time, (days) | $[ISA]_{tot}$, (M) | $R_d$, (L kg$^{-1}$) | Error[a] | Experiment purpose |
|---|---|---|---|---|---|---|
| 38.5 | 5 | 2 | $10^{-2}$ | 130.5 | 3.2 | Sorption kinetics (variation of S/L ratio) |
| 38.5 | 5 | 7 | $10^{-2}$ | 179.3 | 4.4 | |
| 38.5 | 5 | 49 | $10^{-2}$ | 326.9 | 2.9 | |
| 38.5 | 25 | 2 | $10^{-2}$ | 40.76 | 0.76 | |
| 38.5 | 25 | 7 | $10^{-2}$ | 79.5 | 1.5 | |
| 38.5 | 25 | 49 | $10^{-2}$ | 321 | 10 | |
| 38.5 | 50 | 2 | $10^{-2}$ | 42.20 | 0.82 | |
| 38.5 | 50 | 7 | $10^{-2}$ | 72.4 | 1.7 | |
| 38.5 | 50 | 49 | $10^{-2}$ | 386.9 | 6.6 | |
| 20 | 5 | 10 | $10^{-5}$ | 246.0 | 5.4 | Sorption kinetics (variation of S/L ratio and ISA concentration) |
| 20 | 5 | 10 | $10^{-3}$ | 887.3 | 43.7 | |
| 20 | 5 | 10 | $10^{-2}$ | 1155 | 34 | |
| 20 | 5 | 10 | 0.2 | 91.2 | 1.3 | |
| 20 | 25 | 10 | $10^{-5}$ | 180.7 | 8.2 | |
| 20 | 25 | 10 | $10^{-3}$ | 178.4 | 6.6 | |
| 20 | 25 | 10 | $10^{-2}$ | 294.7 | 6.6 | |
| 20 | 25 | 10 | 0.2 | 59.21 | 0.94 | |
| 20 | 5 | 20 | $10^{-2}$ | 349.0 | 9.1 | |
| 20 | 25 | 20 | $10^{-2}$ | 417 | 30 | |
| 20 | 5 | 35 | $10^{-2}$ | 1441 | 58 | |
| 20 | 25 | 35 | $10^{-2}$ | 541 | 48 | |
| 20 | 5 | 70 | $10^{-5}$ | 1036 | 32 | |
| 20 | 5 | 70 | $10^{-3}$ | 913 | 15 | |
| 20 | 5 | 70 | $10^{-2}$ | 2093 | 142 | |
| 20 | 5 | 70 | 0.2 | 45.5 | 2.5 | |
| 20 | 25 | 70 | $10^{-5}$ | 868 | 17 | |
| 20 | 25 | 70 | $10^{-3}$ | 631 | 103 | |
| 20 | 25 | 70 | $10^{-2}$ | 631 | 43 | |
| 20 | 25 | 70 | 0.2 | 71.6 | 3.7 | |
| 20 | 5 | 160 | $10^{-5}$ | 1155 | 40 | |
| 20 | 5 | 160 | $10^{-3}$ | 1975 | 37 | |
| 20 | 5 | 160 | $10^{-2}$ | 1774 | 31 | |
| 20 | 5 | 160 | 0.2 | 85.8 | 1.4 | |
| 20 | 25 | 160 | $10^{-5}$ | 3020 | 438 | |
| 20 | 25 | 160 | $10^{-3}$ | 2013 | 4 | |
| 20 | 25 | 160 | $10^{-2}$ | 792 | 26 | |
| 20 | 25 | 160 | 0.2 | 70.3 | 1.3 | |

[a] Uncertainties are reported as two times the standard deviation (2σ) of mean values.

*Table S4. Distribution coefficients, $R_d$, determined in the sorption experiments for the calcite-NaCl-$^{14}$C system carried out with $[NaCl]_{tot}$ = $10^{-4}$-2 M.*

| Initial activity, ($Bq \cdot mL^{-1}$) | S/L, ($g \cdot L^{-1}$) | Time, (days) | $[NaCl]_{tot}$, (M) | $R_d$, ($L\ kg^{-1}$) | Error[a] |
|---|---|---|---|---|---|
| 20 | 5 | 2 | $10^{-4}$ | 1868 | 87 |
| 20 | 5 | 2 | $3.2 \cdot 10^{-3}$ | 1957 | 48 |
| 20 | 5 | 2 | $3.2 \cdot 10^{-2}$ | 2025 | 60 |
| 20 | 5 | 2 | 0.32 | 1989 | 67 |
| 20 | 5 | 2 | 2 | 2181 | 101 |
| 20 | 25 | 2 | $10^{-4}$ | 851 | 30 |
| 20 | 25 | 2 | $3.2 \cdot 10^{-3}$ | 956 | 33 |
| 20 | 25 | 2 | $3.2 \cdot 10^{-2}$ | 906 | 36 |
| 20 | 25 | 2 | 0.32 | 896 | 17 |
| 20 | 25 | 2 | 2 | 5393 | 251 |
| 20 | 5 | 30 | $10^{-4}$ | 826.0 | 3.9 |
| 20 | 5 | 30 | $3.2 \cdot 10^{-3}$ | 550 | 18 |
| 20 | 5 | 30 | $3.2 \cdot 10^{-2}$ | 356.2 | 5.2 |
| 20 | 5 | 30 | 0.32 | 196.2 | 6.2 |
| 20 | 5 | 30 | 2 | 180.1 | 2.2 |
| 20 | 25 | 30 | $10^{-4}$ | 2968 | 474 |
| 20 | 25 | 30 | $3.2 \cdot 10^{-3}$ | 593 | 16 |
| 20 | 25 | 30 | $3.2 \cdot 10^{-2}$ | 279.6 | 1.9 |
| 20 | 25 | 30 | 0.32 | 166.2 | 8.0 |
| 20 | 25 | 30 | 2 | 939 | 12 |
| 20 | 5 | 60 | $10^{-4}$ | 748 | 16 |
| 20 | 5 | 60 | $3.2 \cdot 10^{-3}$ | 686 | 12 |
| 20 | 5 | 60 | $3.2 \cdot 10^{-2}$ | 1104 | 29 |
| 20 | 5 | 60 | 0.32 | 262.5 | 7.8 |
| 20 | 5 | 60 | 2 | 239.4 | 2.9 |
| 20 | 25 | 60 | $10^{-4}$ | 630 | 24 |
| 20 | 25 | 60 | $3.2 \cdot 10^{-3}$ | 952 | 37 |
| 20 | 25 | 60 | $3.2 \cdot 10^{-2}$ | 443 | 12 |
| 20 | 25 | 60 | 0.32 | 265 | 11 |
| 20 | 25 | 60 | 2 | 342.5 | 9.0 |

[a] Uncertainties are reported as two times the standard deviation (2σ) of mean values.

*Table S5. Distribution coefficients, $R_d$, determined in the sorption experiments for the calcite-NaCl-$^{14}$C-ISA system carried out with $[NaCl]_{tot}$ = $10^{-4}$-2 M and $[ISA]_{tot}$ =$10^{-2}$ M.*

| Initial activity, ($Bq \cdot mL^{-1}$) | S/L, ($g \cdot L^{-1}$) | Time, (days) | $[NaCl]_{tot}$, (M) | $[ISA]_{tot}$, (M) | $R_d$, ($L \cdot kg^{-1}$) | Error[a] |
|---|---|---|---|---|---|---|
| 20 | 5 | 5 | $10^{-4}$ | $10^{-2}$ | 1045 | 19 |
| 20 | 5 | 5 | $3.2 \cdot 10^{-3}$ | $10^{-2}$ | 1138 | 14 |
| 20 | 5 | 5 | $3.2 \cdot 10^{-2}$ | $10^{-2}$ | 1061 | 32 |
| 20 | 5 | 5 | 0.32 | $10^{-2}$ | 1040 | 25 |
| 20 | 5 | 5 | 2 | $10^{-2}$ | 1121 | 24 |
| 20 | 25 | 5 | $10^{-4}$ | $10^{-2}$ | 385 | 11 |
| 20 | 25 | 5 | $3.2 \cdot 10^{-3}$ | $10^{-2}$ | 347 | 11 |
| 20 | 25 | 5 | $3.2 \cdot 10^{-2}$ | $10^{-2}$ | 342.3 | 8.9 |
| 20 | 25 | 5 | 0.32 | $10^{-2}$ | 338.8 | 6.7 |
| 20 | 25 | 5 | 2 | $10^{-2}$ | 486 | 33 |
| 20 | 5 | 17 | $10^{-4}$ | $10^{-2}$ | 1379 | 12 |
| 20 | 5 | 17 | $3.2 \cdot 10^{-3}$ | $10^{-2}$ | 1323 | 29 |
| 20 | 5 | 17 | $3.2 \cdot 10^{-2}$ | $10^{-2}$ | 1273 | 31 |
| 20 | 5 | 17 | 0.32 | $10^{-2}$ | 1178 | 31 |
| 20 | 5 | 17 | 2 | $10^{-2}$ | 1309.92 | 0.22 |
| 20 | 25 | 17 | $10^{-4}$ | $10^{-2}$ | 760 | 16 |
| 20 | 25 | 17 | $3.2 \cdot 10^{-3}$ | $10^{-2}$ | 509 | 13 |
| 20 | 25 | 17 | $3.2 \cdot 10^{-2}$ | $10^{-2}$ | 484 | 12 |
| 20 | 25 | 17 | 0.32 | $10^{-2}$ | 481 | 12 |
| 20 | 25 | 17 | 2 | $10^{-2}$ | 556 | 14 |
| 20 | 5 | 93 | $10^{-4}$ | $10^{-2}$ | 511 | 27 |
| 20 | 5 | 93 | $3.2 \cdot 10^{-3}$ | $10^{-2}$ | 5428 | 144 |
| 20 | 5 | 93 | $3.2 \cdot 10^{-2}$ | $10^{-2}$ | 428.6 | 4.6 |
| 20 | 5 | 93 | 0.32 | $10^{-2}$ | 438 | 18 |
| 20 | 5 | 93 | 2 | $10^{-2}$ | 479 | 11 |
| 20 | 25 | 93 | $10^{-4}$ | $10^{-2}$ | 1125 | 53 |
| 20 | 25 | 93 | $3.2 \cdot 10^{-3}$ | $10^{-2}$ | 581.4 | 7.3 |
| 20 | 25 | 93 | $3.2 \cdot 10^{-2}$ | $10^{-2}$ | 363 | 12 |
| 20 | 25 | 93 | 0.32 | $10^{-2}$ | 245.9 | 5.0 |
| 20 | 25 | 93 | 2 | $10^{-2}$ | 396 | 30 |

[a] Uncertainties are reported as two times the standard deviation (2σ) of mean values.

# 8 References


1. M. F. L'Annunziata, *Handbook of radioactivity analysis*, Academic press, 2012.
2. B. Madé, W. Bower, S. Brassinnes, E. Colàs, L. Duro, P. Blanc, A. Lassin, L. Harvey and J. Begg, *Applied Geochemistry*, 2025, **180**, 106273.
3. M. Ochs, D. Mallants and L. Wang, *Radionuclide and metal sorption on cement and concrete*, Springer, 2016.
